# A novel metric to improve mismatched primer selection and quantification accuracy in amplifying DNA repeats for quantitative polymerase chain reactions

**DOI:** 10.1371/journal.pone.0292559

**Published:** 2023-10-09

**Authors:** Eugenia Y. Xu, Lisa M. Schneper, Daniel A. Notterman

**Affiliations:** Department of Molecular Biology, Princeton University, Princeton, NJ, United States of America; University of Helsinki: Helsingin Yliopisto, FINLAND

## Abstract

In quantitative polymerase chain reaction (qPCR) experiments, primers containing mismatches with respect to the template are widely used in measuring repetitive DNA elements. Primer-template mismatches may lead to underestimation of the input sample quantity due to inefficient annealing and amplification. But how primer-template mismatches affect quantification accuracy has not been rigorously investigated. In this study, we performed a series of qPCR experiments in which we tested three pairs of mismatched telomere primers (tel1/tel2, tel1b/tel2b and telg/telc) and two pairs of perfect-match reference gene primers (36B4-F/-R and IFNB1-F/-R) at three different primer concentrations under four cycling conditions. Templates used were genomic DNA from two human cell lines and oligo duplexes which contained telomere sequences, reference gene sequences, or both. We demonstrated that the underestimation of input sample quantity from reactions containing mismatched primers was not due to lower amplification efficiency (*E*), but due to ineffective usage of the input sample. We defined a novel concept of *amplification efficacy (f)* which quantifies the effectiveness of input sample amplification by primers. We have modified the conventional qPCR kinetic formula to include *f*, which corrects the effects of primer mismatches. We demonstrated that reactions containing mismatched telomere primer pairs had similar efficiency (*E*), but varying degrees of reduced efficacy (*f*) in comparison to those with the perfect-match gene primer pairs. Using the quantitative parameter *f*, underestimation of initial target by telomere primers can be adjusted to provide a more accurate measurement. Additionally, we found that the tel1b/tel2b primer set at concentration of 500 nM and 900 nM exhibited the best amplification efficacy *f*. This study provides a novel way to incorporate an evaluation of amplification efficacy into qPCR analysis. In turn, it improves mismatched primer selection and quantification accuracy in amplifying DNA repeats using qPCR methods.

## Introduction

The quantitative polymerase chain reaction (qPCR) has been used to determine the copy numbers of repetitive DNA elements such as telomere repeats, short interspersed and long interspersed nuclear elements (SINEs and LINEs), as well as the number of copies of a gene of interest in a microbial community of an environmental sample [1–5]. In these qPCR examples, degenerate and mismatched primers are frequently used. Mismatches between primer and template alter duplex stability, thus affecting extension by *Taq* polymerase and leading to reduced amplification of the PCR product [6–8]. A single mismatched base in half of the primer sequence closest to the 3’ end has been shown to result in an underestimation of up to 1,000-fold of the gene copy number [9,10]. Since repetitive DNAs comprise more than 50% of the human genome and are present in most organisms [11–13], improving quantification accuracy when using mismatched primers will help in studying the function of repetitive DNA elements using qPCR methods.

Telomeres are highly conserved, non-coding sequences consisting of tandem repeats of TTAGGG at the ends of vertebrate chromosomes. In human chromosomes, they are 5,000 to 15,000 nucleotides long. Telomeres shorten with each cell division [14]. When telomeres shorten to a critical length, cell senescence occurs [15,16]. Telomere length (TL) has gained popularity as a biomarker; apparently accelerated telomere shortening has been associated with conditions including obesity, diabetes, and cardiovascular disease [17,18]. Additionally, shorter telomere length has been used as a biomarker for exposure to lifetime stress [19–22]. Several methods have been developed to measure TL [23–25]. Among them, the qPCR method is widely used due to its low cost, minimal DNA requirement, quick turnaround, and suitability for high-throughput analyses. However, due to differences in qPCR assay design and measurement, replication has been problematic [26]. This could be due to pre-analytical variables, varied sources of master mixes and technical issues, which have been evaluated in some reports [27–30]. It could also be due to the choices and concentrations of the specific mismatched telomere primers used in qPCR assays. The three sets of telomere primers frequently used to measure TL in the literature, tel1/tel2, tel1b/tel2b and telg/telc, mainly differ in the positioning of mismatched nucleotides with respect to vertebrate telomere repeats [4,31–33]. The annealing/extension efficiency of the three sets of mismatched telomere primers to the telomere repeats may vary. It is worth exploring if telomere primer pairs differing in identity to telomere repeats give similar estimates of initial template quantities when using identical qPCR conditions, including the input template.

Copy number quantification in qPCRs is described by the basic equation for qPCR kinetics, N_*c*_ = N_0_*E^*c*^, which defines the number of copies or quantity of PCR products at *c* cycles (N_*c*_) as the initial number of copies or input sample quantity (N_0_) times the PCR amplification efficiency (*E*) to the power *c* [34–36]. The number of cycles required to reach a fluorescence threshold that is above the background signal, is defined as the quantification cycle (C_q_). C_q_ values are inversely proportional to the amount of starting material in a reaction; the lower the C_q_ value, the greater the amount of the initial target N_0_ [37]. Amplification efficiency (*E*) is defined as the ratio of the number of target gene molecules at the end of a PCR cycle (*c+1*) divided by the number of target molecules at the start of the same PCR cycle (*c*) and expressed as *E =* N_*c+1*_/N_*c*_. Under the optimal condition, amplification efficiency (*E*) is 100%, which is represented as a value of 2. Based on the qPCR kinetic formula, the parameters of threshold fluorescence, C_q_ and *E* are needed to estimate N_0_.

Conventionally, PCR efficiency (*E*) assessment is based on the slope of the standard curve (SC) that is generated from qPCR of a serial dilution of a double stranded template. Specifically, the C_q_ values of the SC are plotted versus the log10 of the template concentrations comprising the SC. The slope is obtained after performing linear regression on the SC. The efficiency can be derived as *E* = 10^(-1/slope)^ [38,39]. The amplification efficiency obtained from the SC method does not represent the PCR efficiency of each reaction on the same plate due to sample differences, concentrations, and technical issues [40,41]. In order to calculate the amplification efficiency of each reaction using amplification curves from each well, several algorithms have been developed [42–47]. These algorithms are developed based on the conventional PCR kinetic equation N_*c*_ = N_0_*E^*c*^. Specifically, the LinRegPCR program is based on log-linearizing the basic equation resulting in Log(N_c_) = Log(N_0_) + C*Log(E) [45]. The LinRegPCR program evaluates patterns of fluorescence during the exponential phase on a reaction-by-reaction basis [45,48,49]. The linear range of the exponential phase is determined by the automatic setting of a window-of-linearity (W-o-L) based on the fluorescence data collected in a qPCR assay. Linear regression analysis is used to calculate the intercept and the slope. From the linear regression line Log(N_0_) = —C*Log(E) + LogN_c_, the initial target quantity N_0_ = 10^intercept^ and PCR efficiency *E* = 10^slope^ can be estimated. The advantage of the LinRegPCR program is that it provides the initial target quantity N_0_ accounting for the mean amplification efficiency of each amplicon, C_q_, and *E* of each reaction in the experiment, which is convenient for quantification and analysis.

It has been reported that amplification efficiency (*E*) of mismatched primers is lower than that of perfect-match primers because the annealing of mismatched primers to the template and elongation by *Taq* polymerase is less efficient [50,51]. However, primer-template mismatch mainly happens in the first two cycles of a qPCR assay, which results in fewer target molecules at the start of cycle three. From cycle three, the mismatched primers are perfectly matched to the newly synthesized amplicons from the first two cycles, and PCR products may double with each cycle under optimal conditions. A standard qPCR amplification curve contains three distinct phases: baseline phase, exponential phase, and plateau phase [38]. During the exponential phase, which is often after cycle three, the amount of PCR product approximately doubles in each cycle. Since qPCR analysis algorithms generally inspect the fluorescence during the exponential phase to determine the C_q_ and the amplification efficiency (*E*), the amplification efficiency (*E*) even with mismatched primers can be close to 100%. Thus, the quantification bias from mismatched primers is not due to or indicated by lower amplification efficiency (*E*).

The aim of this study is to understand what causes quantification bias and how to improve qPCR quantification accuracy when using mismatched primers. We hypothesize that underestimation of input sample quantity when using mismatched primers is due to ineffective usage of the initial target. Here we utilized three pairs of telomere primers (tel1/tel2, tel1b/tel2b, and telg/telc) as examples of mismatched primers and two sets of primers (36B4-F/-R and IFNB1-F/-R) for human reference genes as examples of perfect-match gene primers. *36B4* encodes the human acidic ribosomal phosphoprotein P0 (*RPLP0*) and *IFNB1* encodes the human interferon beta 1 gene. We used templates consisting of oligo duplexes containing either 84 bp of 14 telomere repeats TTAGGG, 75 bp from the *36B4* gene, or 83 bp from the *IFNB1* gene. We also used two oligo duplex templates containing both telomere and either *36B4* or *IFNB1* reference gene sequences. Additionally, we used genomic DNA from two human cell lines as template. We demonstrated that telomere primers tel1/tel2, tel1b/tel2b, and telg/telc exhibited varying degrees of underestimation of N_0_, although the amplification efficiency (*E*) of the five sets of primers was similar. Among the three sets of telomere primers, the tel1b/tel2b primer set showed the most accurate estimation of N_0_ in amplifying telomere repeats using oligo duplexes as templates. This paper provides a novel way to improve mismatched primer selection and quantification accuracy in qPCR experiments.

## Materials and methods

### Reagents

Human cell lines 3C167b [52,53] and NHFpreT [54], referred to as C1 and C3 in this paper, respectively, are immortalized human normal fibroblast cells derived from IMR90 and GM847 cells that were transfected with telomerase (gifts from Dr. Yuanjun Zhao, Pennsylvania State University). C1 and C3 cells were maintained in Dulbecco’s Modified Eagle Medium (DMEM), supplemented with 1x MEM Non-Essential Amino Acids (NEAA), 1x GlutaMAX, 1x sodium pyruvate, 1x Penicillin-Streptomycin (Pen-Strep), and 10% certified Fetal Bovine Serum (FBS). All reagents for cell culture work were ordered from Life Technologies (Carlsbad, CA). All cells were kept in a 37°C humidified incubator with 95% air and 5% CO_2_.

Primers (Table 1) and oligo duplexes (Table 2) were manufactured by Integrated DNA Technologies (Coralville, IA).

**Table 1:** Primer pairs used in this study.

Name (Forward/ reverse primer)	Forward primer sequence (5’–3’)	Reverse primer sequence (5–3’)	Source, reference
tel1/tel2	**ggtttt**(t**g**aggg)_5_t	**tcccga**(cta**t**cc)_5_cta	[4]
tel1b/tel 2b	**cggttt**(gtt**t**gg)_5_gtt	**ggcttg**(cct**t**ac)_5_cct	[31,32]
telg/telc	**acactaa**(ggtt**t**g)_4_ggttag**t**gt	**tgttagg**(ta**t**ccc)_5_taac**a**	[33]
36B4-F/36B4-R	cagcaagtgggaaggtgtaatcc	cccattctatcatcaacgggtacaaac	[4] & this study
IFNB1-F/IFNB1-R	tggcacaacaggtagtaggcgacac	gcacaacaggagagcaatttggagga	[55]

Nucleotides in bold are mismatched to the telomere repeats and all other nucleotides are perfectly matched to their respective templates.

**Table 2:** Oligo duplexes used as templates for qPCR experiments.

Name	Oligomer sequence (5’–3’)	Source, reference
Tel-ds	**(TTAGGG)**_**14**_	
Tel-36B4-ds	**(TTAGGG)**_**14**_CAGCAAGTGGGAAGGTGTAATCCGTCTCCACAGACAAGGCCAGGACTCGTTTGTACCCGTTGATGATAGAATGGGttt	This study
Tel-IFNB1-ds	**(TTAGGG)**_**14**_gcacaacaggagagcaatttggaggagacacttgttggtcatgttgacaacacgaacagtgtcgcctactacctgttgtgccattt	This study
36B4-ds	TTTCAGCAAGTGGGAAGGTGTAATCCGTCTCCACAGACAAGGCCAGGACTCGTTTGTACCCGTTGATGATAGAATGGGAAA	[56] & this study
IFNB1-ds	TTTgcacaacaggagagcaatttggaggagacacttgttggtcatgttgacaacacgaacagtgtc gcctactacctgttgtgccaAAA	[55] & this study
IFNB1-36B4-ds	AAAgcacaacaggagagcaatttggaggagacacttgttggtcatgttgacaacacgaacagtgtcgcctactacctgttgtgccaCAGCAAGTGGGAAGGTGTAATCCGTCTCCACAGACAAGGCCAGGACTCGTTTGTACCCGTTGATGATAGAATGGGttt	This study

Bold and capital letters are for telomere repeats. Capital letters are for the *36B4* reference gene amplicon and small letters are for the *IFNB1* reference gene amplicon, except the first 3Ts, last 3ts or 3As.

### DNA isolation and dilutions

Genomic DNA (gDNA) from human cell lines was extracted using the DNeasy Blood and Tissue Kit (Qiagen Inc., Germantown, MD) following the manufacturer’s instructions. The concentrations of gDNA and oligo duplexes were quantified using the Quant-iT PicoGreen dsDNA Assay Kit (Thermo Fisher Scientific, Waltham, MA) following the manufacturer’s instructions. DNA purity was evaluated using a NanoDrop 2000 spectrophotometer (Thermo Fisher Scientific, Waltham, MA) based on the readings of optical density (OD) at 260 and 280 nanometers (nm).

Template DNA for qPCR experiments was diluted as follows: gDNA was first diluted to 10 ng/μL in water, then further diluted to 2.5 ng/μL. Oligo duplexes were first diluted to 30 pg/μL in water, then further serially diluted 10-fold in six or seven, as noted in the text.

### Quantitative PCR assay

All qPCR assays were carried out on a 384-well plate with adhesive film (Thermo Fisher Scientific, Waltham, MA) using a QuantStudio Flex 6 real-time PCR system (Applied Biosystems, Waltham, MA). The reaction mixture was prepared in a final volume of 10 μL, containing 5 μL of 2x PowerUp Sybr Green Master Mix (Thermo Fisher Scientific, Waltham, MA), 2 μL of template DNA, and 3 μL of primer pairs in water at a final concentration of 100 nM, 500 nM, or 900 nM for respective forward and reverse primers as indicated in the text. Reactions with telomere primers and reference gene primers were performed on the same plate. Each assay contained no-template-control (NTC) wells with all reaction components except that water was added instead of template DNA. All reactions were performed in triplicate. The amplification protocol used one of the following four cycling programs (Table 3). All experiments were repeated independently at least three times.

**Table 3:** qPCR cycling programs and protocols.

Cycling protocol	Cycling program			
	#1 (60°C only)	#2 (49°C-60°C)	#3 (56°C-60°C)	#4 (60°C-60°C)
Stage 1–Hold	50°C for 2 min and 95°C for 10 min			
Stage 2–PCR for two cycles		2 cycles of 95°C for 15 sec, 49°C for 1 min	2 cycles of 95°C for 15 sec, 56°C for 1 min	2 cycles of 95°C for 15 sec, 60°C for 1 min
Stage 3–PCR with fluorescence collection	40 cycles of 95°C for 15 sec, 60°C for 1 min, with fluorescence signal collection			
Stage 4–Melt curve	1 cycle of 95°C for 15 sec, 60°C for 1 min, 95°C for 15 sec, with fluorescence signal collection			

Data acquisition was performed with the QuantStudio real-time PCR software v1.7.2 (Thermo Fisher Scientific, Waltham, MA).

### Analysis of amplification data

Raw data was exported and uploaded to the LinRegPCR website (https://www.gear-genomics.com/rdml-tools/linregpcr.html) [48] for analysis by the LinRegPCR program. The exported parameters after analysis included PCR efficiency *E*, threshold cycle number C_q_, and initial target quantity N_0_.

### Statistical analysis

All statistical analyses and graphs were performed using GraphPad Prism software, version 9.4.1 (GraphPad Software, San Diego, CA). The statistical significance of ratio of amplification efficacy between two groups and of PCR efficiency between two groups was analyzed using the unpaired *t*-test with Welch’s correction, and the statistical significance of PCR efficiency among three or more groups was analyzed using Brown-Forsythe and Welch ANOVA tests. *p* < 0.05 was considered significant.

## Results

### Similar amplification efficiency (*E*) between mismatched and perfect-match primers

Three pairs of telomere primers (tel1/tel2, tel1b/tel2b, and telg/telc) that differ in the

position of non-complementary nucleotides relative to telomere repeats are widely used to measure telomere length (TL) by qPCR in human, mouse, and other vertebrate samples [4,28,31,33]. The amplification efficiencies of the three sets of primers in amplifying telomere repeats have not been compared side-by-side. We amplified identical quantities of telomere repeats under identical cycling conditions to analyze the amplification efficiency (*E*) and the initial target quantity (N_0_) of reactions containing each of the telomere primer pairs at 100 nM, 500 nM, and 900 nM concentrations. We tested six 10-fold serial dilutions of the telomere template (Tel-ds), an 84 bp oligo duplex containing 14 telomere repeats (TTAGGG), with the highest quantity being 60 picograms (pg). As a parallel comparison, we also tested six 10-fold serial dilutions of oligo duplexes containing either the 75 bp human *36B4* gene fragment (36B4-ds) or the 83 bp human *IFNB1* gene fragment (IFNB1-ds), starting with 6 pg. These were used as templates for perfect-match gene primers. Each serial dilution was amplified by either of its corresponding telomere (mismatch) or reference gene (perfect-match) primers of the same concentration on the same plate using Cycling Program #1 (60°C only).

**Fig 1:**
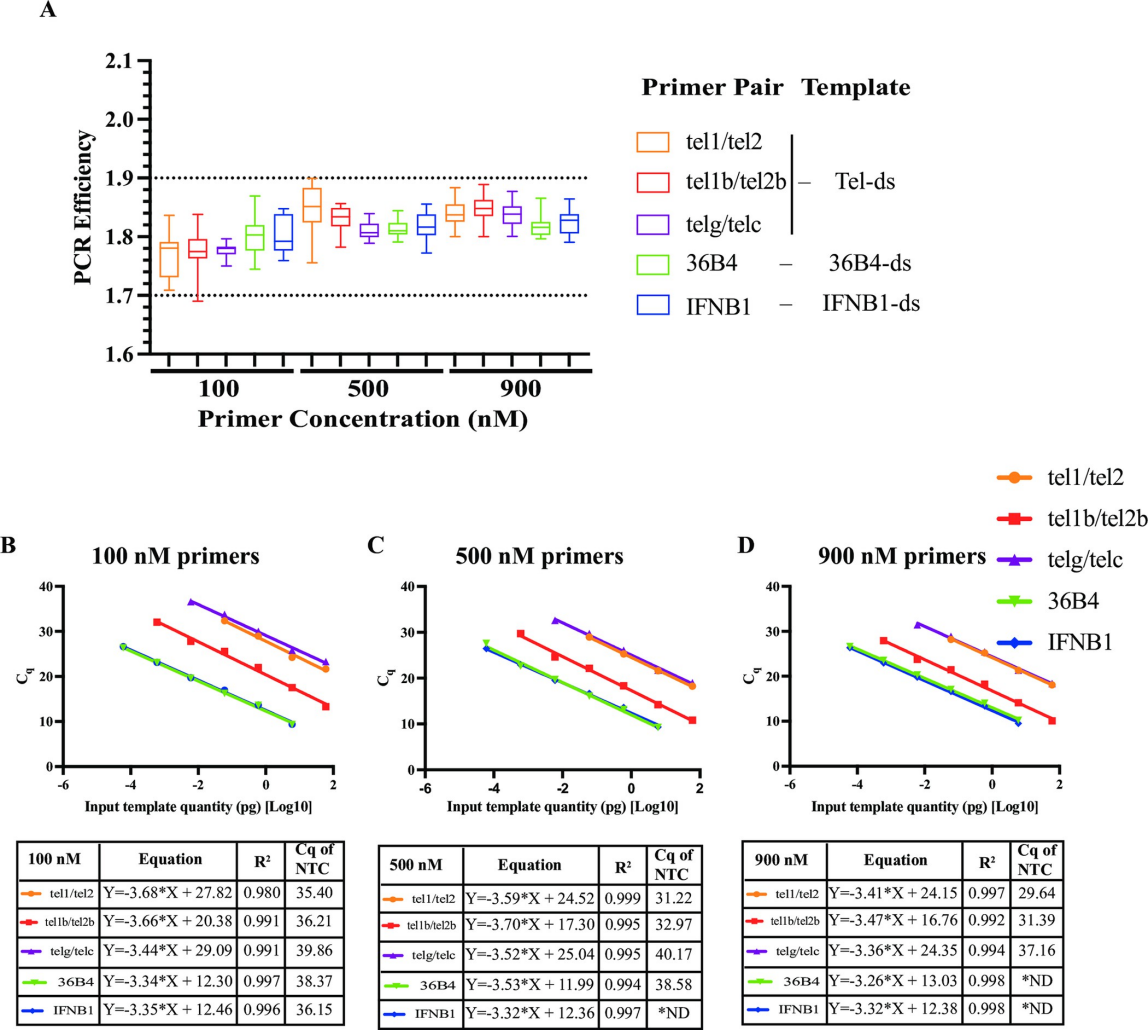
Similar PCR amplification efficiency (*E*) between mismatched telomere and perfect-match reference gene primer pairs with respective oligo duplex templates. **(A).** Individual PCR efficiency from each reaction containing the same primer pair of the same concentration with serial dilutions of the respective oligo duplex templates Tel-ds, 36B4-ds, and IFNB1-ds at 60 or 6, 0.6, 0.06, 0.006, 0.0006 or 0.0006 pg running under Cycling Program #1 (60°C only). The box & whisker plots range from Min to Max and the horizontal line in the box is the median. **B-D.** Standard curves are plotted with C_q_ values of qPCR data in **A** versus Log10 quantity of input template. The parameters corresponding to the curves are shown in the tables below each figure with corresponding primers of 100 nM (**B**), 500 nM (**C**) and 900 nM (**D**). *ND: Not Determined. NTC: No-Template-Control.

The PCR efficiency values of all the conditions tested (six serial dilutions of three respective templates amplified with either three mismatched telomere or two perfect-match gene primer pairs at 100 nM, 500 nM, and 900 nM concentrations) ranged from 1.69 to 1.90 (Fig 1A). This range is consistent with previous reports of PCR efficiency when analyzed using the LinRegPCR program [38,57,58]. The median efficiencies of the five different primer pairs were very close when comparing reactions containing the same primer concentration, with 100 nM ranging from 1.77 to 1.80, 500 nM ranging from 1.81 to 1.85, and 900 nM ranging from 1.82 to1.85 (Fig 1A). Statistical tests were performed on the *E* values that were used to plot Fig 1A. The tests showed that PCR efficiencies for reactions between the two sets of perfect-match gene primer pairs were not significantly different, and PCR efficiencies among the three sets of mismatched telomere primers of the same concentration were not significantly different at 100 nM and 900 nM but significantly different at 500 nM (S1 Table). Regardless, the mean *E* values among the five sets of primer pairs of the same concentration were similar (S1 and S2 Tables).

We next evaluated C_q_ and initial target quantity N_0_ values in the corresponding reactions containing identical quantities of input template. The LinRegPCR program uses the average *E* values of the amplicon on the same plate to estimate N_0_ values for each reaction containing the same primer pair. In this case, the *E* values of each amplicon were closer. An example of the data from reactions containing 6 pg input template is shown in Table 4A. Lower C_q_ values were correlated with higher N_0_ values, as expected. The estimated initial target quantities (N_0_) from the two reference genes at any tested primer concentration were similar with 0.67- to 1.48-fold differences using identical quantities of respective input templates, indicating that similar PCR efficiencies were correlated with similar C_q_ and N_0_ values between the two perfect-match primer pairs (Table 4A and S2 Table), as expected. However, when using identical quantities of different input templates for reactions with perfect-match and mismatched primer pairs, the estimated N_0_ values for reactions containing the mismatched telomere primer pairs were much lower than the reference genes N_0_, ranging from 13- to 150-fold, 953- to 7445-fold, and 968- to 25651-fold less for tel1b/tel2b, tel1/tel2, and telg/telc primers of any tested concentration, respectively (Table 4A and S2 Table). The trend of N_0_ differences between reactions containing perfect-match gene primer pairs and mismatched telomere primer pairs was not correlated with slightly higher or lower PCR efficiency of corresponding reactions, suggesting that underestimation of N_0_ of reactions containing mismatched telomere primer pairs was not due to lower PCR efficiencies. In addition, with similar PCR efficiencies among the three pairs of mismatched telomere primers, the estimated N_0_ values were greatest from reactions containing the tel1b/tel2b primer pair when using identical Tel-ds input template. Tel1b/tel2b N_0_ values were 47- to 93-fold more than those from reactions containing the tel1/tel2 primer pair, and 63- to 171-fold more than those containing the telg/telc primer pair at any tested concentration. This supported that underestimation of N_0_ from reactions containing mismatched telomere primer pairs was not due to lower PCR efficiencies and average PCR efficiency from reactions containing mismatched telomere primer pairs and perfect-match gene primer pairs was similar.

**Table 4:** An example of N_0_, C_q_ and *E* values from qPCRs containing 6 pg input template using Cycling Program #1 (60°C only). A. An example of the output parameters of qPCR data when analyzed using the LinRegPCR program. B. N_0_ and *E* values calculated using the traditional method–*E* from the standard curves and N_0_ from basic PCR kinetic formula.

**Primer concentration (nM)**		**100**			**500**			**900**		
**Template**	**Primer pair**	**N**_**0**_	**C**_**q**_	***E***	**N**_**0**_	**C**_**q**_	***E***	**N**_**0**_	**C**_**q**_	***E***
Tel-ds	tel1/tel2	2.03 x 10^−7^	24.38	1.77	8.29 x 10^−7^	21.64	1.83	9.33 x 10^−7^	21.27	1.84
	tel1b/tel2b	9.63 x 10^−6^	17.59	1.78	7.75 x 10^−5^	14.16	1.83	7.60 x 10^−5^	13.99	1.84
	telg/telc	6.73 x 10^−8^	25.81	1.79	9.52 x 10^−7^	21.75	1.81	9.30 x 10^−7^	21.38	1.83
36B4-ds	36B4	8.84 x 10^−4^	9.36	1.82	1.67 x 10^−3^	9.17	1.82	9.62 x 10^−4^	10.01	1.83
IFNB1-ds	IFNB1	9.66 x 10^−4^	9.33	1.80	1.50 x 10^−3^	9.28	1.82	1.23 x 10^−3^	9.56	1.83
**Primer concentration (nM)**		**100**			**500**			**900**		
**Template**	**Primer pair**	**N**_**0**_	**C**_**q**_	***E***	**N**_**0**_	**C**_**q**_	***E***	**N**_**0**_	**C**_**q**_	***E***
Tel-ds	tel1/tel2	5.64 x 10^−8^	24.38	1.87	3.69 x 10^−7^	21.64	1.90	2.43 x 10^−7^	21.27	1.96
	tel1b/tel2b	3.68 x 10^−6^	17.59	1.88	6.09 x 10^−5^	14.16	1.86	3.76 x 10^−5^	13.99	1.94
	telg/telc	7.34 x 10^−9^	25.81	1.95	2.74 x 10^−7^	21.75	1.92	1.82 x 10^−7^	21.38	1.98
36B4-ds	36B4	3.72 x 10^−4^	9.36	1.99	1.01 x 10^−3^	9.17	1.92	3.34 x 10^−4^	10.01	2.03
IFNB1-ds	IFNB1	3.90 x 10^−4^	9.33	1.99	6.40 x 10^−4^	9.28	2.00	5.30 x 10^−4^	9.56	2.00

N_0_ and C_q_ are median values from triplicate wells. *E* are average *E* values of all reactions containing the primer pair on the same plate.

To further evaluate PCR efficiency (*E*) and the estimated initial target quantity (N_0_) from reactions with perfect-match and mismatched primer pairs, we employed the traditional standard curve (SC) method to calculate *E* = 10^(-1/slope)^ based on the slope from the SCs and N_0_ = N_c_/*E*^*c*^ with the above data. Standard curves were plotted with the C_q_ values from reactions of each primer pair versus Log10 of input template quantity (Fig 1B–1D). Because the C_q_ values of wells with 0.006 or 0.0006 pg Tel-ds were higher than those from the no-template-control (NTC) wells using tel1/tel2 or telg/telc primer pairs, these points were omitted from the SCs for tel1/tel2 and telg/telc primer pairs. Only four or five points, instead of six points, were depicted in these two curves. PCR efficiencies calculated from the traditional method (Table 4B) were higher than those from the LinRegPCR program, as reported [38,57]. Consistent with the above results analyzed using the LinRegPCR program, the traditional SC method confirmed that reactions containing either mismatched or perfect-match primer pairs had similar PCR efficiencies (*E*) (Table 4B), especially at 500 nM and 900 nM. The estimated N_0_ values of each reaction from the traditional method were mostly about 1.27- to 3.84-fold lower than those from the LinRegPCR program because of slightly higher PCR efficiencies derived from the SC method (Table 4A and 4B). Regardless of slightly higher or lower PCR efficiency, the N_0_ values calculated from the traditional method were greatly reduced in reactions with mismatched telomere tel1/tel2 and telg/telc primer pairs (3–4 orders of magnitude) and the tel1b/tel2b primer pair (ranging from 9- to 106-fold) with respect to those with perfect-match gene primer pairs; and the N_0_ values from reactions containing the perfect-match gene primer pairs were roughly the same (ranging from 0.63- to 1.57-fold), in line with the LinRegPCR program results (Table 4A and 4B). The relative N_0_ differences from reactions containing either of the perfect-match gene primer pairs and those containing each of the mismatched telomere primer pairs were roughly in the same range using both methods, indicating the traditional method and the LinRegPCR program had similar estimation in N_0_ values.

Then, we asked whether a different qPCR software, like the qPCR instrument software—QuantStudio Real-time PCR software (QuantStudio software), would provide similar *E* and N_0_ values when analyzing the same qPCR data. We analyzed the above qPCR data using QuantStudio software with set threshold at 0.2 and automatic threshold. The two sets of C_q_ values were used to generate respective SCs as seen in S1A–S1C Fig for set threshold and Fig 1D–1F for automatic threshold. The SCs from the two sets of C_q_ values were basically the same. *E* and N_0_ values calculated from the traditional method (*E* = 10^(-1/slope)^ based on the slope from the SCs and N_0_ = N_c_/*E*^*c*^) were the same as well (S3A and S3B Table), which were like those obtained using C_q_ values from the LinRegPCR program (Table 4B). This further supported the above observations when analyzed using the LinRegPCR program.

Together, all the analysis methods demonstrated that reactions with mismatched telomere primer pairs and perfect-match reference gene primer pairs had similar amplification efficiencies (*E*), but that the mismatched primer pairs underestimated the initial template quantity greatly. Furthermore, this suggested that only a fraction of the starting template was utilized by the mismatched telomere primers in qPCRs. Since the QuantStudio software and the LinRegPCR program gave similar C_q_ values, and *E* and N_0_ values calculated from the traditional SC method and from the LinRegPCR program did not produce meaningful differences in the analysis, we only report N_0_ and *E* values exported from the LinRegPCR program. The LinRegPCR program provides the initial target quantity N_0_, C_q_, and *E* for each reaction, which is convenient for quantification and analysis.

### qPCR amplification efficacy (*f*)

The major difference in the qPCR kinetics between mismatched and perfect-match primers to their respective targets lies in the first two amplification cycles. During the first two cycles, inefficient hybridization of the mismatched primers to the starting template affects extension by the *Taq* DNA polymerase, so the initial template quantity does not increase two-fold in each cycle as is expected. Beginning in cycle three, the qPCR kinetics of mismatched primers is approximately equal to that of perfect-match primers since the mismatched primers become perfectly matched to the newly synthesized targets from the first two cycles. Because qPCR analysis programs inspect the fluorescence above the background signal, the basic equation for qPCR kinetics is the same for mismatched and perfect-match primers. As stated in [35,58], the basic equation for qPCR kinetics is:

$$N_{c}=N_{0}E^{c}$$ (1)

In this equation, N_*c*_ is the number of target copies after *c* cycles, N_0_ is the estimated number of initial target molecules or initial quantity, and *E* is the PCR amplification efficiency. It is assumed that N_0_ is the actual quantity or copy number of the template added to the reaction, and that all template molecules can be recognized by the primers and the primers extended by the *Taq* DNA polymerase. This is true for the perfect-match primers such as 36B4 and IFNB1 reference gene primers. However, from the qPCR analysis using the mismatched telomere primers, we observed that only a fraction of the starting material was used for amplification in qPCR. To account for this phenomenon, we propose a novel concept of amplification efficacy, referred to as *f*, which quantifies the effectiveness of input template amplification in a qPCR assay. It is defined as

$$f=\frac{N_{0}}{N_{i}}$$ (2)

Here, N_0_ is the observed quantity of the initial target. N_i_ is the actual quantity of the input sample that is added to the well. The amplification efficacy (*f*) can be represented as a value between 0 and 1, with 1 being 100% efficacy, which is the case for perfect-match primers under ideal reaction conditions. By including the amplification efficacy (*f*) parameter in Eq 1, we have adapted the conventional qPCR kinetic formula to account for both perfect-match and mismatched primers. Eq 1 is modified as follows:

$$N_{c}={fN}_{i}E^{c}$$ (3)

For perfect-match primers, the observed number of copies of the initial target equals to the actual number of copies of the input sample; N_0_ is equal to N_i_, and the amplification efficacy *f* is equal to 1. Eq 3 becomes Eq 1, which is a special case for Eq 3.

If the quantity of input sample N_i_ is the same for two sets of primer pairs

(${N_{i}}_{\left(primer\;pair\;1\right)}={N_{i}}_{\left(primer\;pair\;2\right)}$), the ratio of amplification efficacy between the two sets of primer pairs can be derived from Eq 2 as follows:

$$\mathrm{ratio}\;\mathrm{of}\;\mathrm{amplification}\;\mathrm{efficacy}=\frac{f_{\left(primer\;pair\;1\right)}}{f_{(primer\;pair\;2)\;}}=\frac{{N_{0}}_{\left(primer\;pair\;1\right)}}{{N_{0}}_{(primer\;pair\;2)\;}}$$ (4)

Here, N_0(primer pair 1)_ and N_0(primer pair 2)_ values are the estimated N_0_ for the respective primer pairs from the LinRegPCR program developed based on Eq 1 [45,48,49]. The value of ratio of amplification efficacy indicates if primer pair 1 has better (value greater than 1), worse (value less than 1) or the same (value equal to 1) than primer pair 2. This can help select optimal mismatched primers for qPCRs.

### Reduced amplification efficacy (*f*) from mismatched primers

To directly compare the effectiveness of the mismatched telomere primers with perfect-match reference gene primers in qPCR amplification, we designed two oligo duplexes containing 84 bp telomere repeats and either 75 bp from the *36B4* gene (Tel-36B4-ds), or 83 bp from the *IFNB1* gene (Tel-IFNB1-ds). Using these templates, the quantity of the starting material for the mismatched telomere primers and perfect-match reference gene primers is ensured to be identical. An oligo duplex template containing 83 bp from the *IFNB1* gene and 75 bp from the *36B4* gene (IFNB1-36B4-ds) was used as a control. The IFNB1-36B4-ds template was used to ensure the perfect-match primers IFNB1 and 36B4 indeed amplified the identical template with similar amplification efficiency (*E*) and amplification efficacy (*f*). Since reactions with telomere primer pairs and reference gene primer pairs are run in separate wells and the two sets of primer pairs do not compete for the availability of the input sample, this design of oligo duplexes with telomere repeats and one reference gene amplicon sequences or two reference gene amplicon sequences does not impact availability of input templates. Serial dilutions of the three oligo duplexes (at 6, 0.6, and 0.06 pg) were used as templates for the experiments. We tested the primer concentrations of 100 nM, 500 nM, and 900 nM in qPCR experiments with Cycling Program #1 (60°C only).

**Fig 2:**
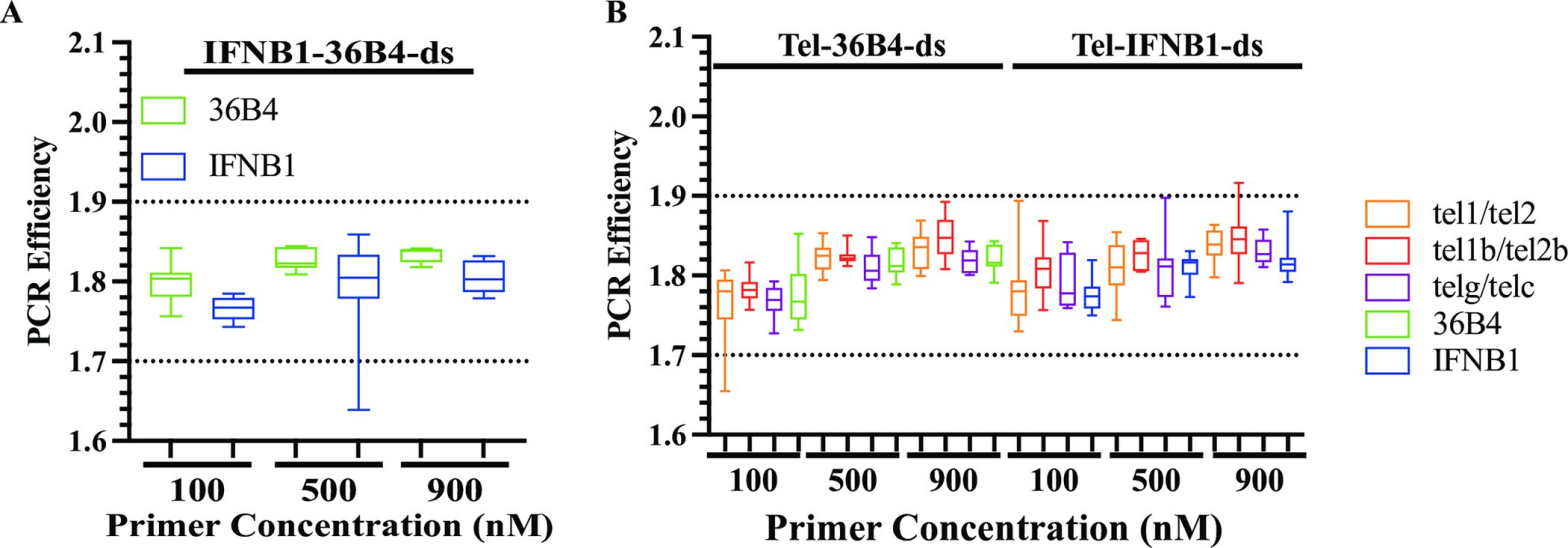
Similar PCR amplification efficiency (*E*) between mismatched telomere and perfect-match reference gene primers with identical oligo duplex template. Individual PCR efficiency from each reaction containing serial dilutions of respective oligo duplex templates at 6, 0.6 and 0.06 pg using Cycling Program #1 (60°C only). (**A).** 36B4 and IFNB1 primer pairs with oligo duplex IFNB1-36B4-ds as template. (**B).** The five sets of primer pairs with oligo duplexes Tel-36B4-ds and Tel-IFNB1-ds as templates.

We first examined the qPCR data from template IFNB1-36B4-ds. The two reference gene primers showed amplification efficiencies (*E*) ranging from 1.64 to 1.86 with similar median *E* for the same primer concentrations (ranging from 1.77 to 1.80 for 100 nM, 1.80 to 1.82 for 500 nM, and 1.80 to 1.84 for 900 nM) (Fig 2A). The two perfect-match gene primer pairs showed similar mean amplification efficiencies (*E*) (S1 and S2 Tables), as expected. When identical quantities of input IFNB1-36B4-ds template were used, the estimated N_0_ values were roughly the same for the reactions using either of the perfect-match reference gene primer pairs at any primer concentration tested (Cycling Program #1 (60°C only) in Table 5 and S2 Table). Based on Eq 4, the ratios of the amplification efficacy (*f*_(36B4)_/*f*_(IFNB1)_) between the two reference gene primers were around one, ranging from 0.85 to 1.43, with identical quantities of the input template, indicating that the amplification efficacy (*f*) between the two perfect-match reference gene primer pairs was roughly the same with about 100% efficacy. This confirmed that an oligo duplex template with two-amplicon sequences can be used to compare the amplification efficacy (*f*) and efficiency (*E*) between the two primer sets.

**Table 5:** Estimated N_0_ for reactions containing reference gene primer pairs 36B4 and IFNB1 using the IFNB1-36B4-ds oligo duplex template.

Cycling program		#1 (60°C only)		#2 (49°C-60°C)		#3 (56°C-60°C)		#4 (60°C-60°C)	
Primer concentration (nM)	Template quantity (pg)	36B4	IFNB1	36B4	IFNB1	36B4	IFNB1	36B4	IFNB1
100	6	1.86 x 10^−3^	1.78 x 10^−3^	5.81 x 10^−3^	5.32 x 10^−3^	6.72 x 10^−3^	6.18 x 10^−3^	5.19 x 10^−3^	5.33 x 10^−3^
	0.6	1.29 x 10^−4^	1.42 x 10^−4^	4.65 x 10^−4^	4.34 x 10^−4^	5.42 x 10^−4^	5.58 x 10^−4^	4.32 x 10^−4^	4.62 x 10^−4^
	0.06	1.84 x 10^−5^	2.16 x 10^−5^	6.98 x 10^−5^	6.51 x 10^−5^	7.86 x 10^−5^	8.27 x 10^−5^	5.82 x 10^−5^	6.28 x 10^−5^
500	6	2.68 x 10^−3^	2.04 x 10^−3^	6.28 x 10^−3^	5.85 x 10^−3^	5.58 x 10^−3^	4.57 x 10^−3^	6.36 x 10^−3^	5.78 x 10^−3^
	0.6	2.47 x 10^−4^	1.84 x 10^−4^	4.81 x 10^−4^	4.50 x 10^−4^	4.02 x 10^−4^	3.38 x 10^−4^	5.65 x 10^−4^	5.03 x 10^−4^
	0.06	2.88 x 10^−5^	2.01 x 10^−5^	6.79 x 10^−5^	6.93 x 10^−5^	5.14 x 10^−5^	4.96 x 10^−5^	7.02 x 10^−5^	6.77 x 10^−5^
900	6	2.23 x 10^−3^	2.08 x 10^−3^	8.58 x 10^−3^	7.17 x 10^−3^	7.01 x 10^−3^	6.22 x 10^−3^	7.89 x 10^−3^	6.83 x 10^−3^
	0.6	1.83 x 10^−4^	1.47 x 10^−4^	8.28 x 10^−4^	7.07 x 10^−4^	5.91 x 10^−4^	5.24 x 10^−4^	7.65 x 10^−4^	6.64 x 10^−4^
	0.06	2.08 x 10^−5^	1.82 x 10^−5^	8.82 x 10^−5^	7.10 x 10^−5^	6.19 x 10^−5^	5.47 x 10^−5^	8.86 x 10^−5^	7.31 x 10^−5^

N_0_ are median values from triplicate wells.

Next, we compared the amplification efficiency (*E*) and amplification efficacy (*f*) of telomere primers with reference gene primers by performing qPCR experiments as described above with serial dilutions of oligo duplexes Tel-36B4-ds and Tel-IFNB1-ds as templates. All reactions with 100 nM, 500 nM, and 900 nM primer concentrations showed PCR efficiency ranging from 1.65 to 1.92 (Fig 2B). The median *E* values were most similar when the primer pair concentrations were the same, ranging from 1.77 to 1.81, 1.81 to 1.83, and 1.81 to 1.85 at 100 nM, 500 nM, and 900 nM, respectively. The mean *E* values from these five sets of primer pairs exhibited similar amplification efficiencies (*E*) (S1 and S2 Tables). Then, we used Eq 4 to calculate the ratio of amplification efficacy of telomere primers relative to 36B4 primers *f*_(tel)_/*f*_(36B4)_ for the Tel-36B4-ds template and the ratio of amplification efficacy of telomere primers relative to IFNB1 primers *f*_(tel)_/*f*_(IFNB1)_ for the Tel-IFNB1-ds template. Indeed, the three pairs of telomere primers showed varying degrees of reduced amplification efficacy (*f*) relative to the reference gene primers in amplifying both oligo duplexes (Cycling Program #1 (60°C only) in Table 6). The estimated N_0_ values for 36B4 or IFNB1 primers in amplifying identical quantities of respective oligo duplex were similar at any tested primer concentration (Cycling Program #1 (60°C only) in Table 7), suggesting that the amplification efficacy of the reference gene primers was around 100% or 1, as expected. So, the higher ratio of amplification efficacy (*f*_(tel)_/*f*_(36B4)_ or *f*_(tel)_/*f*_(IFNB1)_) is consistent with the better amplification efficacy of the pair of telomere primers. The ratios of amplification efficacy (*f*_(tel)_/*f*_(36B4)_ and *f*_(tel)_/*f*_(IFNB1)_) were lowest in reactions containing 100 nM telomere primers for all three template concentrations tested, while the efficacy was similar in reactions containing 500 nM or 900 nM primers, with the efficacy being slightly higher in reactions with 900 nM primers. This suggested that the telomere primers were rate-limiting at the concentration of 100 nM and that of the concentrations tested, at least 500 nM should be used. Among the three sets of telomere primers using either oligo duplex as template, the primer set tel1b/tel2b demonstrated the best efficacies that were about 20-fold less than that of reference gene primers at concentrations of 900 nM or 500 nM, while primer sets tel1/tel2 and telg/telc had roughly similar amplification efficacies that were approximately 1370- to 2182-fold lower than that of reference gene primers at primer concentrations of 900 nM and 500 nM. Since amplification efficiencies (*E*) of the mismatched telomere primers and the perfect-match gene primers were similar, the amplification efficacy (*f*) parameter helped quantify the effectiveness of mismatched primers using the input template in qPCR amplification.

**Table 6:** Summary of ratios of amplification efficacy (*f*_(tel)_/*f*_(36B4)_ or *f*_(tel)_/*f*_(IFNB1)_).

Primer concentration (nM)	Template	Tel-36B4-ds			Tel-IFNB1-ds		
	Cycling program	$\frac{f_{\left(tel1\right)}}{f_{\left(36B4\right)}}$	$\frac{f_{\left(tel1b\right)}}{f_{\left(36B4\right)}}$	$\frac{f_{\left(telg\right)}}{f_{\left(36B4\right)}}$	$\frac{f_{\left(tel1\right)}}{f_{\left(IFNB1\right)}}$	$\frac{f_{\left(tel1b\right)}}{f_{\left(IFNB1\right)}}$	$\frac{f_{\left(telg\right)}}{f_{\left(IFNB1\right)}}$
100	#1 (60°C only)	1.36 x 10^−4^	8.35 x 10^−3^	7.42 x 10^−5^	1.08 x 10^−4^	6.63 x 10^−3^	3.58 x 10^−5^
	#2 (49°C-60°C)	1.83 x 10^−3^	5.02 x 10^−2^	2.20 x 10^−3^	3.52 x 10^−3^	6.18 x 10^−2^	2.40 x 10^−3^
	#3 (56°C-60°C)	1.52 x 10^−3^	1.57 x 10^−1^	3.88 x 10^−3^	2.12 x 10^−3^	1.31 x 10^−1^	3.61 x 10^−3^
	#4 (60°C-60°C)	2.63 x 10^−4^	1.00 x 10^−2^	5.80 x 10^−5^	1.26 x 10^−4^	7.45 x 10^−3^	2.88 x 10^−5^
500	#1 (60°C only)	5.11 x 10^−4^	4.48 x 10^−2^	5.88 x 10^−4^	5.93 x 10^−4^	4.82 x 10^−2^	4.58 x 10^−4^
	#2 (49°C-60°C)	3.63 x 10^−3^	9.56 x 10^−2^	5.93 x 10^−3^	5.42 x 10^−3^	1.10 x 10^−1^	4.64 x 10^−3^
	#3 (56°C-60°C)	4.78 x 10^−3^	3.73 x 10^−1^	2.47 x 10^−2^	5.20 x 10^−3^	3.59 x 10^−1^	1.48 x 10^−2^
	#4 (60°C-60°C)	7.45 x 10^−4^	6.18 x 10^−2^	7.50 x 10^−4^	6.73 x 10^−4^	5.22 x 10^−2^	4.83 x 10^−4^
900	#1 (60°C only)	7.30 x 10^−4^	5.04 x 10^−2^	6.95 x 10^−4^	5.31 x 10^−4^	5.06 x 10^−2^	4.77 x 10^−4^
	#2 (49°C-60°C)	6.40 x 10^−3^	1.35 x 10^−1^	9.50 x 10^−3^	9.56 x 10^−3^	1.60 x 10^−1^	6.46 x 10^−3^
	#3 (56°C-60°C)	7.20 x 10^−3^	4.58 x 10^−1^	4.67 x 10^−2^	9.90 x 10^−3^	3.97 x 10^−1^	2.34 x 10^−2^
	#4 (60°C-60°C)	1.12 x 10^−3^	1.17 x 10^−1^	1.14 x 10^−3^	8.86 x 10^−4^	8.50 x 10^−2^	6.45 x 10^−4^

*f*_(tel)_/*f*_(36B4)_ or *f*_(tel)_/*f*_(IFNB1)_ are median values between telomere primer pairs tel1/tel2 (tel1), telb/tel2b (tel1b), telg/telc (telg), and reference gene primer pairs 36B4 and IFNB1 from all reactions containing 6, 0.6, and 0.06 pg Tel-36B4-ds or Tel-IFNB1-ds templates, respectively.

**Table 7:** Estimated N_0_ for reactions containing reference gene primer pairs using 6, 0.6 and 0.06 pg oligo duplex template.

Primer concentration (nM)	Primer pair	36B4			IFNB1		
	Template	Tel-36B4-ds			Tel-IFNB1-ds		
	Cycling program	6	0.6	0.06	6	0.6	0.06
100	#1 (60°C only)	9.45 x 10^−4^	5.81 x 10^−5^	9.36 x 10^−6^	1.68 x 10^−3^	1.42 x 10^−4^	2.10 x 10^−5^
	#2 (49°C-60°C)	2.94 x 10^−3^	2.34 x 10^−4^	3.79 x 10^−5^	5.33 x 10^−3^	4.98 x 10^−4^	7.58 x 10^−5^
	#3 (56°C-60°C)	3.85 x 10^−3^	2.83 x 10^−4^	4.86 x 10^−5^	6.16 x 10^−3^	5.42 x 10^−4^	8.03 x 10^−5^
	#4 (60°C-60°C)	3.49 x 10^−3^	2.60 x 10^−4^	4.05 x 10^−5^	5.67 x 10^−3^	5.63 x 10^−4^	9.05 x 10^−5^
500	#1 (60°C only)	1.26 x 10^−3^	7.13 x 10^−5^	9.51 x 10^−6^	1.60 x 10^−3^	1.88 x 10^−4^	2.33 x 10^−5^
	#2 (49°C-60°C)	2.26 x 10^−3^	1.22 x 10^−4^	1.68 x 10^−5^	4.50 x 10^−3^	5.38 x 10^−4^	7.02 x 10^−5^
	#3 (56°C-60°C)	1.63 x 10^−3^	8.43 x 10^−5^	1.29 x 10^−5^	3.54 x 10^−3^	4.08 x 10^−4^	5.40 x 10^−5^
	#4 (60°C-60°C)	3.10 x 10^−3^	1.86 x 10^−4^	2.45 x 10^−5^	4.72 x 10^−3^	5.85 x 10^−4^	7.92 x 10^−5^
900	#1 (60°C only)	9.16 x 10^−4^	5.68 x 10^−5^	8.79 x 10^−6^	1.95 x 10^−3^	1.76 x 10^−4^	2.42 x 10^−5^
	#2 (49°C-60°C)	1.28 x 10^−3^	1.22 x 10^−4^	3.33 x 10^−5^	3.98 x 10^−3^	6.57 x 10^−4^	7.96 x 10^−5^
	#3 (56°C-60°C)	1.01 x 10^−3^	1.03 x 10^−4^	2.53 x 10^−5^	3.59 x 10^−3^	5.65 x 10^−4^	6.23 x 10^−5^
	#4 (60°C-60°C)	1.28 x 10^−3^	1.38 x 10^−4^	3.25 x 10^−5^	4.26 x 10^−3^	6.64 x 10^−4^	7.95 x 10^−5^

N_0_ are median values from triplicate wells.

### Improved amplification efficacy of mismatched primers by lowering the annealing/extension temperature for the first two cycles

We hypothesize that underestimation of input sample quantity from mismatched primers is due to *ineffective* usage of the input template, mainly during the first two qPCR cycles. Beginning in cycle 3, a perfectly matched primer/template duplex is present. Cycling Program #1 (60°C only) in the experiments above used an annealing/extension temperature of 60°C for 40 cycles. A possible reason that telomere primers showed reduced amplification efficacy could be due to inefficient annealing and extension of the mismatched telomere primers at 60°C for the first two cycles. To test our hypothesis, we lowered the annealing/extension temperature for the first two cycles of the qPCR to 49°C (Cycling Program #2 (49°C-60°C)) and 56°C (Cycling Program #3 (56°C-60°C)) to improve annealing/elongation of the telomere primers to telomere repeats. Starting from cycle three, it is expected that the reaction wells would contain newly synthesized amplicons from the first two cycles that would have sequences that were perfectly complementary to the primers. Thus, after the first two cycles, the annealing/extension temperature was raised to 60°C for 40 additional cycles, during which the fluorescence was measured. For a parallel comparison, we also performed qPCRs with an annealing/extension temperature of 60°C for the first two cycles (Cycling Program #4 (60°C-60°C)), followed by 40 cycles with an annealing/extension temperature of 60°C with signal acquisition. The amplification efficiency (*E*) and efficacy (*f*) were evaluated using these cycling conditions, different primer concentrations (100 nM, 500 nM, and 900 nM), and serial dilutions (6, 0.6, and 0.06 pg) of the IFNB1-36B4-ds, Tel-36B4-ds, and Tel-IFNB1-ds oligo duplex templates.

**Fig 3:**
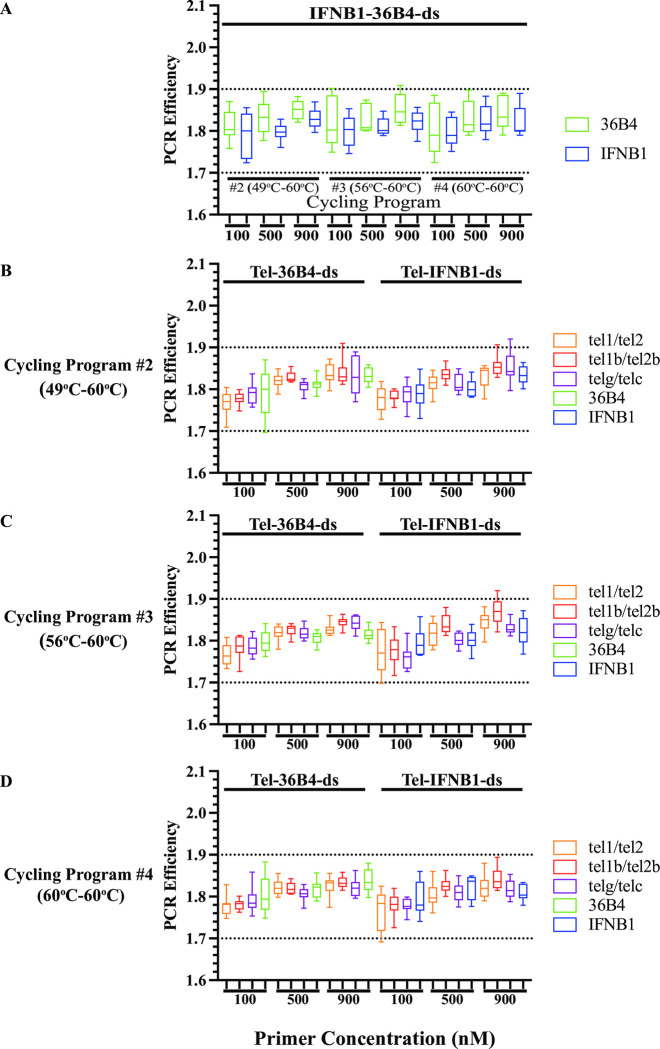
No effects on amplification efficiency (*E*) of mismatched and perfect-match primers with lowered annealing/extension temperatures for the first two cycles. Individual PCR efficiency from each reaction containing serial dilutions of the respective oligo duplex templates at 6, 0.6 and 0.06 pg. **(A).** Reactions containing 36B4 and IFNB1 primer pairs with oligo duplex IFNB1-36B4-ds template using Cycling Program #2 - #4. **B-D.** Reactions containing the five sets of primer pairs with oligo duplexes Tel-36B4-ds and Tel-IFNB1-ds as templates running on Cycling Program #2 (49°C-60°C) (**B**), #3 (56°C-60°C) (**C**) and #4 (60°C-60°C) (**D**).

We first examined the PCR efficiency of reactions from the three cycling programs for reference gene primers with the oligo duplex IFNB1-36B4-ds template to understand whether lowering the annealing/extension temperature for the first two cycles had any effects on amplification from perfect-match gene primers. The PCR efficiency for all the reactions ranged from 1.72 to 1.91, with median *E* between 1.79 and 1.80, 1.80 and 1.83, and 1.80 and 1.85 for the respective 100nM, 500 nM and 900 nM primer pair concentrations (Fig 3A). These resembled the PCR efficiency observed using Cycling Program #1 (60°C only) (Fig 2A) and showed that the mean *E* for the reference gene primers was similar under the studied conditions (S1 and S4 Tables). We then examined the estimated N_0_ from three dilutions of 6, 0.6, and 0.06 pg IFNB1-36B4-ds templates. The N_0_ values from the two reference gene primer pairs were approximately the same at any primer concentration tested using any of the cycling conditions with identical quantities of template (Cycling Program #2–#4 in Table 5). The ratios of amplification efficacy (*f*_(36B4)_/*f*_(IFNB1)_) between the two reference gene primers were around one, ranging from 0.93 to 1.24 under the conditions analyzed, indicating that the two perfect-match reference gene primer pairs had similar amplification efficacies (*f*) with about 100% efficacy. Thus, lowering annealing/extension temperatures for the first two cycles did not affect the amplification of perfect-match gene primers.

We next evaluated the PCR efficiency of reactions from the three cycling programs for telomere and reference gene primers with oligo duplex templates Tel-36B4-ds and Tel-IFNB1-ds. Of the experimental conditions tested, all reactions from the two oligo duplexes showed PCR efficiencies ranging from 1.69 to 1.92 with median *E* from 1.76 to 1.80, 1.80 to 1.84, and 1.80 to 1.87 for primer concentrations of 100 nM, 500 nM, and 900 nM, respectively (Fig 3B–3D). The mean *E* from the three telomere primer pairs and the two reference gene primer pairs also had similar amplification efficiencies (*E*) using these modified cycling conditions (S1 and S4 Tables), and these were similar to those previously observed using Cycling Program #1 (60°C only) (Fig 2B).

We then calculated the ratios of the amplification efficacy of telomere primers versus reference gene primers from the experiments using serial dilutions of oligo duplexes Tel-36B4-ds and Tel-IFNB1-ds as templates. Consistent with our hypothesis, lowering the annealing/extension temperature from 60°C to 49°C or 56°C for the first two cycles improved the ratios of telomere/reference gene amplification efficacy (*f*_(tel)_/*f*_(36B4)_ or *f*_(tel)_/*f*_(IFNB1)_) under any studied conditions (Cycling Program #2 or #3 vs. #4 in Table 6). We performed statistical tests on the values of amplification efficacy from reactions using Cycling Program #2 or # 3 versus #4. The tests showed significant improvement at all tested conditions except for one condition with 900 nM tel1b/tel2b primer pair using Cycling Program #2 (S5 Table). The increase was greatest for the telg/telc primer set (ranging from 8- to 126-fold), followed by the tel1/tel2 primer set (ranging from 5- to 28-fold), and slightly increased for the tel1b/tel2b primer set (ranging from 1.15- to 18-fold). For both reference gene primer pairs, the observed N_0_ values were similar across all conditions (Cycling Program #2–#4 in Table 7), corresponding to the same quantities of starting material, indicating that the improved ratios of amplification efficacy (*f*_(tel)_/*f*_(36B4)_ or *f*_(tel)_/*f*_(IFNB1)_) were due to improved amplification by telomere primers during the first two cycles. This suggested that lowering the annealing/extension temperature for the first two cycles improved usage of the input template by telomere primers. In line with our observations discussed above for qPCRs with Cycling Program #1 (60°C only), the primer set tel1b/tel2b continued to display the best amplification efficacy among the three sets of telomere primers under all experimental conditions. The optimal telomere amplification efficacy observed using 500 nM and 900 nM tel1b/tel2b primers and an annealing temperature of 56°C for the first two cycles were still about two- to three-fold less than those observed for the reference gene primer pairs (Cycling Program #3 in Table 6). Thus, lowering the annealing/extension temperature for the first two cycles improved the amplification by telomere primers, supporting our hypothesis that *inefficient* amplification from mismatched primers was due to *ineffective* usage of input samples.

### Selection of optimal mismatched primer pair based on the values of amplification efficacy (*f*)

**Fig 4:**
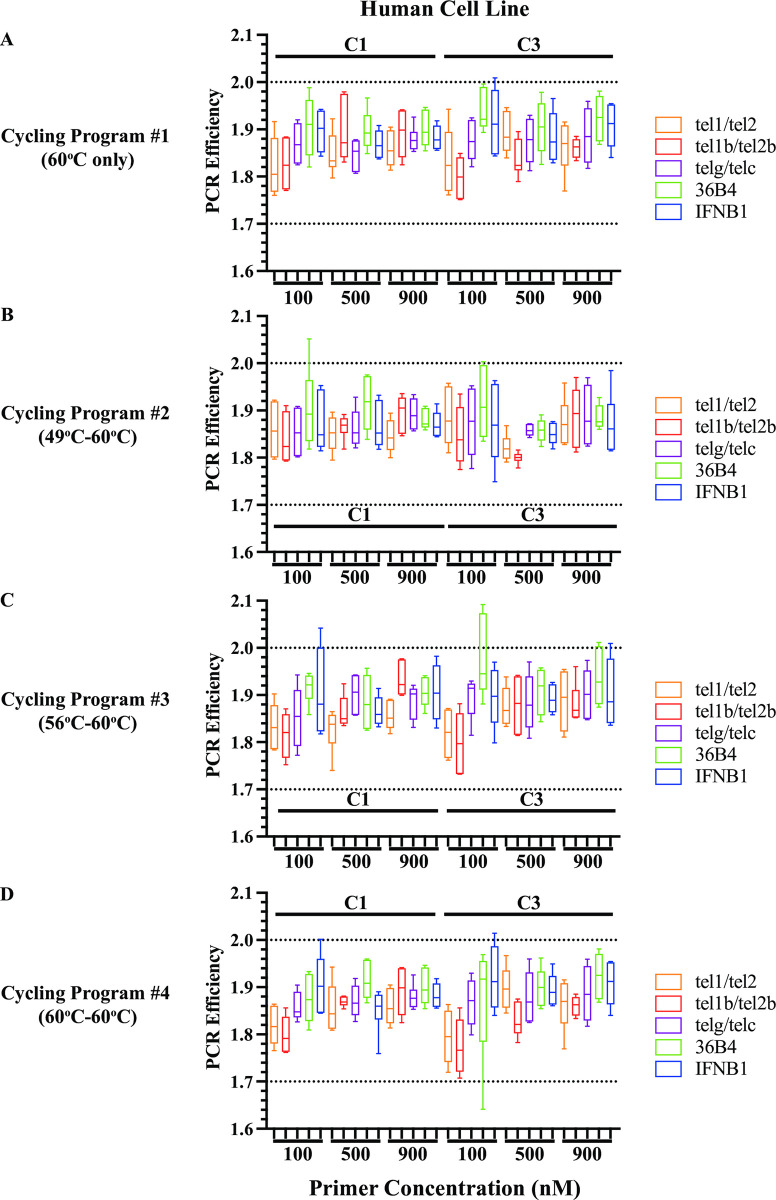
Similar amplification efficiency (*E*) between mismatched telomere and perfect-match reference gene primers using gDNA (5 and 20 ng) of human cell lines as template. Individual PCR efficiency from each reaction containing the same primer pair of the same concentration with 5 and 20 ng gDNA from human cell lines C1 and C3 using Cycling Program #1 (60°C only) (**A)**, #2 (49°C-60°C) (**B)**, #3 (56°C-60°C) (**C**), and #4 (60°C-60°C) (**D**).

In the experiments above using synthetic oligo duplex templates, we showed that although primers with mismatches exhibited similar amplification efficiencies (*E*) with perfect-match primers, they had reduced qPCR amplification efficacies (*f*). To extend this finding to genomic DNA (gDNA) templates, we performed the qPCR experiments described above using 20 ng and 5 ng of gDNA extracted from two human cell lines reported to have short (C1) and long (C3) telomeres [20] as templates. As described in previous sections, we used the five primer pairs (three telomere and two reference gene primer pairs) at three concentrations (100 nM, 500 nM, and 900 nM), and four cycling conditions. We first examined the PCR efficiency (*E*) of all the reactions with human gDNA as templates. Across all experimental conditions, the PCR efficiencies ranged from 1.64 to 2.09 (Fig 4A–4D). The median *E* for the telomere primer pairs, tel1/tel2, tel1b/tel2b, and telg/telc, ranged from 1.80 to 1.90, 1.77 to 1.92, and 1.85 to 1.91, respectively. The median *E* for the reference gene primer pairs ranged from 1.86 to 1.95 for 36B4 and between 1.85 and 1.91 for IFNB1. The mean *E* from the five sets of primer pairs showed similar values as well (S1 Table). This supported the finding using synthetic oligo duplex templates that mismatched telomere primers and perfect-match reference gene primers had similar amplification efficiencies (*E*) in all the conditions tested.

**Fig 5:**
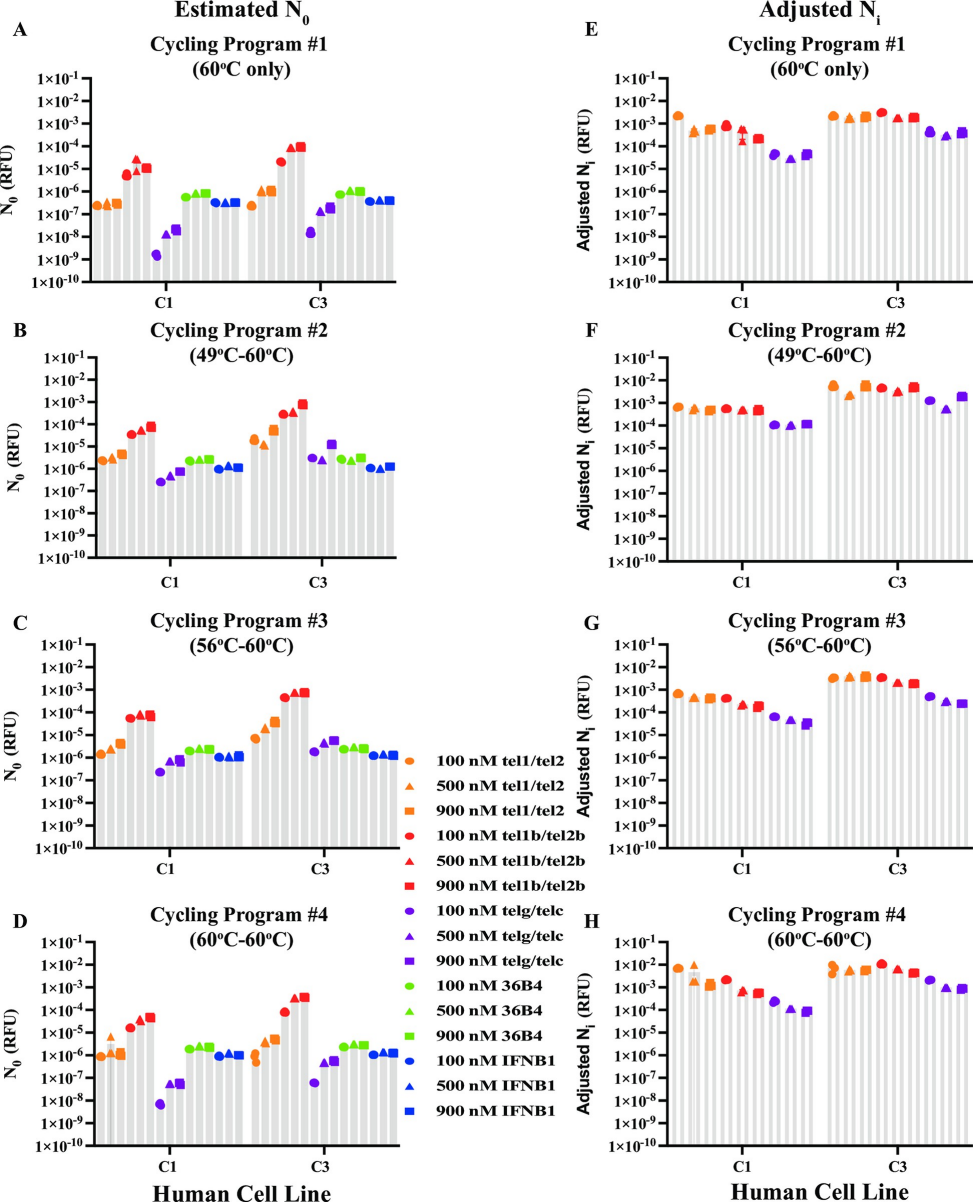
Estimated N_0_ and adjusted N_i_ of the input template quantity using 20 ng gDNA from human cell lines as template. **A-D**. Estimated N_0_ from reactions running under Cycling Program #1 (60°C only) (**A**), #2 (49°C-60°C) (**B**), #3 (56°C-60°C) (**C**), and #4 (60°C-60°C) (**D**). **E-H**. Adjusted N_i_ for telomere measurements from respective qPCR data in **A-D** with Cycling Program #1 (60°C only) (**E**), #2 (49°C-60°C) (**F**), #3 (56°C-60°C) (**G**) and #4 (60°C-60°C) (**H**). Error bars are mean ± SD.

Next, we examined the estimated N_0_ of the two reference genes with the perfect-match 36B4 and IFNB1 primer pairs for each condition tested with gDNA (5 ng and 20 ng) from human cell lines C1 and C3 as templates (Fig 5A–5D and S6 Table). At all the tested conditions, the estimated N_0_ of the *36B4* and the *IFNB1* gene amplicons were about the same between the C1 and C3 cell lines, respectively, when comparing reactions in which the same amount of template had been added. This supports the notion that the copy numbers of the respective reference genes *36B4* and *IFNB1* are the same in human cell lines. Lowering the annealing/extension temperature for the first two cycles in qPCRs with Cycling Program #2 (49°C-60°C) and #3 (56°C-60°C) had no effect on the estimation of N_0_ for respective *36B4* and *IFNB1* genes in both cell lines under any tested conditions compared to that estimated from Cycling Program #4 (60°C-60°C) (Fig 5B–5D and S6 Table). In all experimental conditions tested, the N_0_ values for *36B4* gene amplicons were about two- to three-fold greater than that for *IFNB1* gene amplicons, consistent with the presence of multiple processed pseudogenes of *36B4* throughout the human genome [59]. Together, these results indicated that the two perfect-match reference gene primers had similar amplification efficacies (*f*) under all the conditions tested using gDNA from human cell lines C1 and C3 as templates.

Next, we evaluated the N_0_ estimated from reactions containing the three telomere primer pairs, which were used to quantify telomeres in the C1 and C3 cell lines. The N_0_ estimates were greater for the C3 gDNA template than that for the C1 gDNA template for all conditions tested except for the reactions containing 100 nM tel1 and tel2 primer pair that were amplified using Cycling Program #1 (60°C only) and #4 (60°C-60°C) (Fig 5A–5D and S6 Table). This indicated that the C3 TL is longer than the C1 TL, as was previously described [20]. At a concentration of 100 nM, the tel1/tel2 primer pair may be rate-limiting in the qPCRs to amplify long telomeres with Cycling Program #1 and #4. However, the magnitudes of N_0_ estimated from the three pairs of telomere primers in the same experimental condition differed greatly, although the input sample quantity from each cell line was identical. Lowering the annealing/extension temperature for the first two cycles in qPCRs with Cycling Program #2 (49°C-60°C) and #3 (56°C-60°C) increased the N_0_ estimated from reactions containing tel1/tel2 primers 1.60–20-fold, tel1b/tel2b primers 1.04–6-fold, and telg/telc primers 5–49-fold when compared to the N_0_ estimated from identical reactions that were amplified using Cycling Program #4 (60°C-60°C) instead. Thus, lowering the annealing/extension temperature for the first two cycles improved the usage of the input sample. These data, in which human gDNA was used as templates, were consistent with the findings using oligo duplex templates and further supported our hypothesis that underestimation of the initial target was due to *ineffective* usage of the input sample by mismatched primers.

We then compared the amplification efficacies (*f*) of the three telomere primer pairs. Based on Eq 2, when the N_i_ (input sample quantity) was identical for the three pairs of telomere primers, the amplification efficacies (*f*) of the telomere primers were positively correlated with the estimated N_0_. The highest N_0_ values were observed in reactions containing the tel1b/tel2b primer pair and the lowest with the telg/telc primer pair for all corresponding conditions (Fig 5A–5D and S6 Table). Thus, the tel1b/tel2b primer pair exhibited the best amplification efficacy (*f*) for telomere repeats in gDNA from human cell lines as well as oligo duplexes. Of all the concentrations tested for the three pairs of telomere primers, 500 nM and 900 nM primer concentrations showed better amplification efficacy (*f*) than 100 nM, especially for the long telomeres with human cell line C3. This indicated that the parameter amplification efficacy (*f*) helped select optimal mismatched telomere primers for qPCR experiments.

### Correction of quantification bias in human cells using the amplification efficacy (*f*) obtained with oligo duplex template

In Fig 5A–5D and S6 Table, the N_0_ estimated from the reactions using tel1b/tel2b primers was higher than that estimated from the reactions using the reference gene primers at any condition tested. However, the N_0_ estimated from the reactions using the tel1/tel2 and telg/telc primers were about equal to or less than that estimated from the reactions using the reference gene primers. From this result, it would follow that the numbers of telomere amplicons amplified by telomere primers tel1/tel2 and telg/telc were equal to or less than the reference gene copy numbers in the gDNA of human cell lines C1 and C3. This is not true since a diploid human cell has 92 telomere ends with an average TL between 5,000 and 15,000 base pairs and one to four copies of the reference genes IFNB1 or 36B4. This substantial underestimation can be explained by the much lower amplification efficacies (*f*) of telomere sequences by the tel1/tel2 and telg/telc telomere primer pairs. To correct this quantification bias, we explored whether inclusion of primer amplification efficacy (*f*), derived from experiments using the oligo duplex template containing both telomere and reference gene sequences, could adjust the estimated N_0_ to reflect a more accurate N_i_. Eq 2 would become:

$${N_{i}}_{(\mathrm{telomere}\;\mathrm{primer}\;\mathrm{set})‐\mathrm{gDNA}}=\frac{{N_{0}}_{(\mathrm{telomere}\;\mathrm{primer}\;\mathrm{set})‐\mathrm{gDNA}}}{f_{(telomere\;primer\;set)-gDNA\;}}$$ (5)

Here, N_i_, N_0,_ and *f* values are from reactions containing a pair of telomere primers and gDNA from the C1 or C3 human cell lines. To obtain *f*_(telomere primer set)-gDNA_, we made two assumptions here. The first assumes that *f*_(telomere primer set)-gDNA_ is the same as that obtained from a standard oligo duplex template (*f*_(telomere primer set)-oligo duplex_). Our experiments with oligo duplexes Tel-36B4-ds and Tel-IFNB1-ds as templates showed that the amplification efficacies (*f*) of the three sets of telomere primers of the same concentration were similar for both templates (Table 6). This suggested that the amplification efficacy (*f*) of each primer pair may be template independent. The second assumption is that *f*_(reference gene primer set)_ is 1. This is supported by the observation that the N_0_ values estimated from reactions containing the perfect-match 36B4 or IFNB1 reference gene primer pairs of any concentration using identical quantities of the input template were about the same under respective Cycling Program #1 or #2–#4 (Tables 5 and 7). Thus, the ratios of amplification efficacy of telomere primers relative to 36B4 (*f*_(tel)_/*f*_(36B4)_) or IFNB1 (*f*_(tel*)/*_*f*_(IFNB1)_) primers for each experimental condition using the respective oligo duplex Tel-36B4-ds or Tel-IFNB1-ds template can be considered as the amplification efficacy of telomere primers (*f*_(tel)-oligo duplex_) at each tested condition (Table 6). Incorporating the *f*_(tel)-oligo duplex_ into Eq 5 at each tested condition, we have:

$$\;{N_{i}}_{(\mathrm{telomere}\;\mathrm{primer}\;\mathrm{set})‐\mathrm{gDNA}}=\frac{{N_{0}}_{(\mathrm{telomere}\;\mathrm{primer}\;\mathrm{set})‐\mathrm{gDNA}}}{f_{(telomere\;primer\;set)-oligo\;duplex\;}}$$ (6)

Based on Eq 6, a more accurate N_i_ from each pair of telomere primers in human cell lines C1 and C3 can be calculated from the estimated N_0_ of the corresponding experimental conditions.

In Table 6, the amplification efficacy (*f*_(tel)_) relative to 36B4 or IFNB1 primers obtained from the respective oligo duplex Tel-36B4-ds or Tel-IFNB1-ds was roughly similar for the corresponding telomere primers, indicating it was reference gene-independent. Thus, we used the *f*_(tel)_ generated using the Tel-IFNB1-ds template of the same experimental condition to calculate an adjusted N_i_ since *IFNB1* is a single-copy reference gene in the human genome. For each tested condition, the amplification efficacy (*f*_(tel)_) of each telomere primer set from reactions containing the oligo duplex Tel-IFNB1-ds (Table 6) was used in Eq 6 to calculate the adjusted initial target quantity (adjusted N_i_) for human cell lines C1 and C3 (Fig 5E–5H and S7 Table). Indeed, the adjusted N_i_ for telomere measurements in C1 or C3 human cell line from each pair of telomere primers in each experimental condition was similar or much closer to the identical input sample quantity. The estimated N_0_ for telomere measurements from the tel1/tel2 primer pair was about 13- to 127-fold less than that for the tel1b/tel2b primer pair, and the adjusted N_i_ from both primer pairs was about the same, exhibiting 0.30- to 2-fold differences under any condition for both C1 and C3 cell lines. The estimated N_0_ for telomere measurements from the telg/telc primer pair was about 63- to 3595-fold less than that for the tel1b/tel2b primer pair, and the difference of the adjusted N_i_ for both primer pairs was reduced to a range from 3- to 20-fold under any conditions for both C1 and C3 cell lines. Altogether, inclusion of the amplification efficacy (*f*) obtained from the oligo duplex template helped improve the accuracy of TL measurements in human cell lines.

## Discussion

Effective primers in qPCRs are essential to improving quantification measurements and analysis. The use of degenerate and mismatched primers to measure repetitive DNA elements in qPCRs is often necessary. Mismatched primers amplify the input template less efficiently, leading to an underestimation of the input sample quantity. In this study, we demonstrated that ineffective usage of the input template by mismatched primers resulted in substantial underestimation of initial target quantity. To quantify the effectiveness of input template amplification by mismatched primers in qPCRs, we defined the novel concept of amplification efficacy (*f*) as $f=\frac{N_{0}}{N_{i}}$. With inclusion of the parameter amplification efficacy (*f*), we modified the conventional qPCR kinetic formula to N_*c*_ = *f*N_i_*E*^*c*^ to represent perfect-match and mismatched primers more accurately. Using oligo duplexes Tel-36B4-ds and Tel-IFNB1-ds as templates in systematic qPCR experiments, we discovered that the three pairs of mismatched telomere primers (tel1/tel2, tel1b/tel2b, and telg/telc) had similar PCR amplification efficiencies (*E*), but varying degrees of reduced amplification efficacies (*f*) in comparison to the two pairs of perfect-match reference gene primers (36B4 and IFNB1). The quantitative parameter *f* helped select optimal mismatched primers for qPCRs. With the parameter *f* obtained from reactions containing oligo duplex templates, the underestimated initial quantities of template DNA from the gDNA of human cell lines were adjusted to similar values, which provided a more accurate measurement of the input template.

It has been reported that mismatched primers showed lower amplification efficiencies (*E*) than the perfect-match gene primers in qPCR assays [41,50,51]. However, our comprehensive study indicated that the PCR efficiencies among the three pairs of mismatched telomere primers and two pairs of perfect-match reference gene primers were similar under any of the tested conditions. Statistical tests showed that differences between primer pairs across three primer concentrations were very small and only occasionally significant. The following three pieces of evidence from this study support the statement. The first piece of evidence comes from the study with two pairs of perfect-match reference gene primers 36B4 and IFNB1. In theory, the higher amplification efficiency (*E*) should be correlated with the higher estimated N_0_ when comparing primer pairs using identical quantities of the input template in qPCRs. With identical quantities of the oligo duplex IFNB1-36B4-ds as templates, the estimated N_0_ values from reactions with 36B4 and IFNB1 primers at any tested concentration were roughly the same despite the slight differences in PCR efficiencies among the reactions (ranging from 1.77 to 1.85) (Figs 2A and 3A, and Table 5). In parallel, with gDNA from human cell lines C1 and C3 as templates, although the PCR efficiencies varied from 1.86 to 1.95 for the 36B4 primer pair and from 1.85 to 1.91 for the IFNB1 primer pair, the estimated N_0_ values were about the same for the respective reference gene templates at any primer concentration despite slight differences in *E* (Figs 4 and 5A–5D, and S6 Table). In qPCR experiments with identical quantities of oligo duplex and gDNA as templates, slightly higher PCR efficiencies for the reactions containing 36B4 or IFNB1 primer pairs were not always correlated with slightly higher N_0_. This indicated that the median PCR efficiency, ranging from 1.77 to 1.95, was comparable. Thus, similar PCR efficiencies correlated with similar N_0_ values for the reactions containing perfect match 36B4 and IFNB1 primer pairs in qPCRs, as expected.

The second piece of evidence comes from the study with three pairs of mismatched telomere primers using identical quantities of telomere template from gDNA of two human cell lines. Using human gDNA as templates across all experimental conditions, the median *E* for the telomere primer pairs, tel1/tel2, tel1b/tel2b, and telg/telc, ranged from 1.80 to 1.90, 1.77 to 1.92, and 1.85 to 1.91, respectively (Fig 4A–4D). This range of PCR efficiencies from 1.77 to 1.95 was the same as that for the two perfect-match reference gene primers described above, indicating the three mismatched telomere primer pairs had similar PCR efficiencies. We initially expected that the estimated N_0_ from the three mismatched telomere primer pairs would be similar since the identical telomere template was used. In contrast, the estimated N_0_ in reactions that contained the tel1b/tel2b primers was always much greater than that in reactions containing the other two sets of telomere primers, regardless of whether the PCR efficiency for the tel1b/tel2b primers was higher or lower than the other two telomere primer pairs (Fig 5A–4D and S6 Table). Similarly, the estimated N_0_ in reactions containing the tel1/tel2 primers was always greater than that in reactions containing the telg/telc primers although the PCR efficiency for the tel1/tel2 primers was slightly lower than that for the telg/telc primers in almost all tested conditions. Thus, for the reactions with the mismatched telomere primers, similar amplification efficiencies (*E*) were not correlated with similar N_0_ using identical quantities of the input template. This was different from what we observed in perfect-match gene primers. These results pointed out that the amplification efficiency (*E*) was not the correct term to explain underestimation of N_0_ using mismatched primers in qPCRs.

The third piece of evidence comes from the study with three mismatched telomere primer pairs and two perfect-match reference gene primer pairs using identical quantities of oligo duplexes Tel-36B4-ds and Tel-IFNB1-ds as templates. To investigate whether the oligo duplexes containing telomere repeats and reference gene fragment (two-amplicon oligo duplexes) behave the same as those containing either telomere repeats or reference gene fragment (single-amplicon oligo duplexes), we performed qPCRs with the two-amplicon oligo duplexes (Tel-36B4-ds, Tel-IFNB1-ds and IFNB1-36B4-ds), as well as the single-amplicon oligo duplexes (Tel-ds, 36B4-ds and IFNB1-ds) as templates and confirmed that identical quantity of two-amplicon and single-amplicon oligo duplexes gave similar range of estimated values of *E* and N_0_ with corresponding primer pairs (S2 Table). This indicated that two-amplicon oligo duplexes worked as well as single-amplicon oligo duplexes in qPCRs. With oligo duplexes as templates, telomere and reference gene primers at any tested concentration had similar PCR efficiencies (*E*) ranging from 1.77 to 1.85 (Figs 2B and 3B–3D), as described above. Consistent with the observations from the above two pieces of evidence, similar amplification efficiencies (*E*) for the reactions containing the two perfect-match primer pairs at any concentration correlated with similar N_0_ estimates using identical quantities of the input template (Table 7). Despite similar *E* among telomere and reference gene primers at any tested concentration, the three pairs of mismatched telomere primers showed varying magnitudes of underestimated N_0_ in comparison to the two perfect-match primer pairs using identical quantities of the input template. This was because of varying magnitudes of reduced amplification efficacy (*f*) of the mismatched telomere primer pairs in amplifying the identical quantities of the input template (Table 6). These results further support our finding that the amplification efficiencies (*E*) were similar among mismatched and perfect-match primer pairs, but that differences in amplification efficacies (*f*) explained the underestimation of N_0_ from reactions with mismatched primers. In conclusion, the three pieces of evidence demonstrated that quantification bias from mismatched primers was due to *ineffective* usage of the input template in qPCRs.

The absolute TL method [56] has been widely used to measure TL in humans. This method has an underlying but unstated assumption that the amplification efficacy (*f*) is similar between the mismatched telomere primer pair tel1b/tel2b and the perfect match reference gene primer pair. However, when this method was developed, the concept of amplification efficacy (*f*) had not been introduced. Researchers in the field have assumed that if the amplification efficiency (*E*) is similar between telomere primer pair tel1b/tel2b and the reference gene primer pair, then the absolute TL can be calculated based on the known length in base pairs of the standard oligo duplex containing telomere repeats. Our data indicated that although the amplification efficiency (*E*) of the telomere primer pair tel1b/tel2b and the reference gene primer pair was similar, the telomere primer pair tel1b/tel2b had reduced amplification efficacy (*f*) compared to the reference gene primer pair, which could lead to an inaccurate measurement of TL in base pairs if the absolute TL method was used. With the oligo duplex containing both telomere repeats and the reference gene amplicon as a standard template, the estimated N_0_ of the initial telomere template from human cell line gDNA was adjusted to a more accurate estimate (adjusted N_i_) by incorporating the amplification efficacy (*f*) obtained from the standard oligo duplex (Fig 5E–5H). After the adjustment, the qPCR method may be used to measure the absolute TL in base pairs. Here, we assumed that amplification efficacy of the same primer pair is identical when using oligo duplexes or gDNA from human cell lines as templates. This assumption may be assessed with help of nanopore sequencing. Further experiments evaluating the absolute TL method in qPCRs could be designed using nanopore sequencing in combination with the telomere restriction fragment (TRF) methods to confirm the TL measurements in base pairs by qPCR assays.

The relative T/S (telomere repeats vs. single-copy reference gene) method to evaluate TL can always be used without considering amplification efficacy (*f*) [4]. Cawthon designed tel1/tel2 to measure TL by the conventional qPCR method [4] and telg/telc primers by the monochrome multiplex qPCR (MMQPCR) method [33] using optimized respective qPCR conditions. He used relative T/S method in both cases. The cycling conditions described here were not optimized for tel1/tel2 and telg/telc primer pairs. Our data indicated that without optimized qPCR conditions, amplification efficacy could help adjust the biased TL measurements. In any case, primer selection remains critical, especially when comparing small differences in TL among samples. Considering the amplification efficacy (*f*), the effectiveness of the mismatched primers in amplifying the input template can be quantified, which is beneficial for selecting optimal primers. Among the three mismatched telomere primers used here, the tel1b/tel2b primer pair exhibited the best amplification efficacy with oligo duplexes and gDNA from human cell lines as templates under all tested conditions. From our experiments, primer concentrations greater than or equal to 500 nM showed similar amplification efficacies, with 900 nM performing slightly better. The lowest concentration tested, 100 nM, exhibited the worst amplification. This suggests that at least 500 nM telomere primers are needed for measuring TL in qPCRs.

## Conclusion

In summary, we proposed the concept of amplification efficacy (*f*), defined as $f=\frac{N_{0}}{N_{i}}$, and modified the qPCR kinetic formula as N_*c*_ = *f*N_i_*E*^*c*^ to better represent perfect-match and mismatched primer pairs. We found that *inefficient* amplification from mismatched primers was not due to lower amplification efficiency (*E*), but due to the *ineffective* usage of the input template instead. With inclusion of amplification efficacy (*f*), inefficient amplification from mismatched primers can be quantified and adjusted. This will help improve mismatched primer selection and qPCR quantification accuracy. Although we focused on mismatched telomere primers to amplify telomere repeats, it is expected that measurement and adjustment with amplification efficacy (*f*) can improve the quantification accuracy of other qPCR assays that use mismatched primers.

## Supporting information

S1 Fig Plotted standard curves of qPCR data in Fig 1A when analyzed using QuantStudio real-time PCR software. **A-C.** Standard curves are plotted with C_q_ values obtained from the qPCR data when analyzed with set threshold at 0.2 versus Log10 quantity of input template. **D-F.** Standard curves are plotted with C_q_ values obtained from the qPCR data when analyzed with automatic threshold versus Log10 quantity of input template. The parameters corresponding to the curves are shown in the tables below each figure with corresponding primers of 100 nM (**A & D**), 500 nM (**B & E**) and 900 nM (**C & F**).
(TIF)

S1 Table PCR efficiency (mean±SD) of each primer set corresponding to the box and whisker plots in Fig 1A, 2, 3 and 4, and statistical tests of PCR efficiencies for reactions containing the same template with different primer sets at 100 nM, 500 nM or 900 nM, except the group with respective Tel-ds, 36B4-ds and IFNB1-ds templates. (DOCX)

S2 Table PCR efficiency *E* and estimated initial target quantity N_0_ values for reactions using cycling program #1 (60°C only). Values are expressed in mean±SD from triplicate wells. (XLSX)

S3 Table An example of N_0_ and *E* values calculated with the traditional method for reactions containing 6 pg input template using Cycling Program #1 (60°C only). *E* values are calculated from the corresponding standard curves in S1 Fig and N_0_ values are calculated from basic PCR kinetic formula. C_q_ values are median from triplicate wells corresponding to the qPCR data in S1 Fig.
(DOCX)

S4 Table PCR efficiency *E* and estimated initial target quantity N_0_ values for reactions using Cycling Program #2 - #4. Values are expressed in mean±SD from triplicate wells. (XLSX)

S5 Table Statistical tests of ratio of amplification efficacy (mean±SD) of (*f*_(tel)_/*f*_(36B4)_ or *f*_(tel)_/*f*_(IFNB1)_) in Table 6 between reactions using cycling program #2 or #3 in compared to #4. (DOCX)

S6 Table Estimated N_0_ and C_q_ values from qPCR assays using human gDNA as template. (XLSX)

S7 Table Adjusted N_i_ for telomere measurements in human cell lines, corresponding to N_0_ in S6 Table. (XLSX)
