# Supplementary figures and images for: A novel metric to improve mismatched primer selection and quantification accuracy in amplifying DNA repeats for quantitative polymerase chain reactions
Source: PLoS One. 2023 Oct 9;18(10):e0292559. doi: 10.1371/journal.pone.0292559 (PMC10561853; doi:10.1371/journal.pone.0292559)

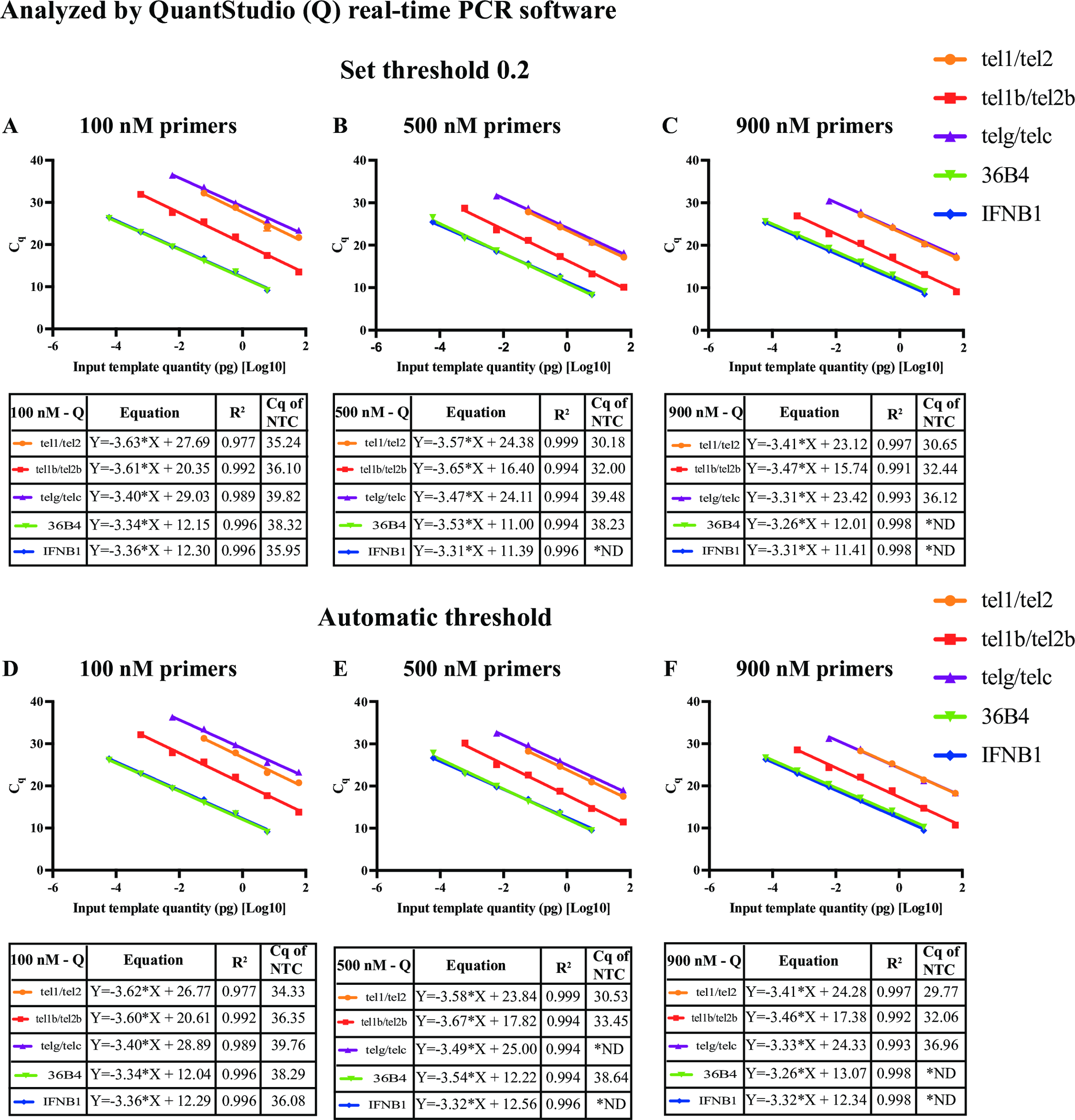

Supplement: S1 Fig — A-C. Standard curves are plotted with Cq values obtained from the qPCR data when analyzed with set threshold at 0.2 versus Log10 quantity of input template. D-F. Standard curves are plotted with Cq values obtained from the qPCR data when analyzed with automatic threshold versus Log10 quantity of input template. The parameters corresponding to the curves are shown in the tables below each figure with corresponding primers of 100 nM (A & D), 500 nM (B & E) and 900 nM (C & F). (TIF) [file pone.0292559.s001.tif]
