# Supplementary material for: A novel metric to improve mismatched primer selection and quantification accuracy in amplifying DNA repeats for quantitative polymerase chain reactions
Source: PLoS One. 2023 Oct 9;18(10):e0292559. doi: 10.1371/journal.pone.0292559 (PMC10561853; doi:10.1371/journal.pone.0292559)
Supplement: S1 Table — (DOCX) [file pone.0292559.s002.docx]

**Supplemental Table 1. PCR efficiency (mean±SD) of each primer set corresponding to the box and whisker plots in Fig 1A, 2, 3 and 4, and statistical tests of PCR efficiencies for reactions containing the same template with different primer sets at 100 nM, 500 nM or 900 nM, except the group with respective Tel-ds, 36B4-ds and IFNB1-ds templates.**

| **Cycling program** | | **#1 (60^o^C only)** | | | **#2 (49^o^C -60^o^C)** | | | **#3 (56^o^C -60^o^C)** | | | **#4 (60^o^C -60^o^C)** | | |
| --- | --- | --- | --- | --- | --- | --- | --- | --- | --- | --- | --- | --- | --- |
| **Primer concentration (nM)** | | **100** | **500** | **900** | **100** | **500** | **900** | **100** | **500** | **900** | **100** | **500** | **900** |
| **Template** | | **Tel-ds** | | |  | | | | | | | | |
| **Primer**  **pair** | tel1/tel2 | 1.77±0.04 | 1.85±0.04 | 1.84±0.02 |  |  |  |  |  |  |  |  |  |
|  | tel1b/tel2b | 1.77±0.04 | 1.83±0.02 | 1.85±0.02 |  |  |  |  |  |  |  |  |  |
|  | telg/telc | 1.78±0.01 | 1.81±0.02 | 1.84±0.02 |  |  |  |  |  |  |  |  |  |
| p-value  among the 3 sets of primers | | 0.9095 | 0.0251 | 0.3355 |  |  |  |  |  |  |  |  |  |
| Significance (<0.05) | | ns | * | ns |  |  |  |  |  |  |  |  |  |
| **Template** | | **36B4-ds** | | |  |  |  |  |  |  |  |  |  |
| **Primer**  **pair** | 36B4 | 1.80±0.03 | 1.81±0.01 | 1.82±0.02 |  |  |  |  |  |  |  |  |  |
| **Template** | | **IFNB1-ds** | | |  |  |  |  |  |  |  |  |  |
| **Primer**  **pair** | IFNB1 | 1.80±0.03 | 1.82±0.02 | 1.83±0.02 |  |  |  |  |  |  |  |  |  |
| p-value  among the 2 sets of primers | | 0.9486 | 0.496 | 0.2554 |  |  |  |  |  |  |  |  |  |
| Significance (<0.05) | | ns | ns | ns |  |  |  |  |  |  |  |  |  |
| **Template** | | **IFNB1-36B4-ds** | | | | | | | | | | | |
| **Primer**  **pair** | 36B4 | 1.80±0.02 | 1.83±0.01 | 1.83±0.01 | 1.81±0.04 | 1.83±0.04 | 1.85±0.02 | 1.82±0.06 | 1.83±0.03 | 1.85±0.04 | 1.81±0.06 | 1.83±0.04 | 1.84±0.04 |
|  | IFNB1 | 1.77±0.01 | 1.79±0.06 | 1.81±0.02 | 1.79±0.05 | 1.80±0.02 | 1.83±0.02 | 1.80±0.04 | 1.81±0.02 | 1.82±0.03 | 1.80±0.03 | 1.83±0.04 | 1.82±0.03 |
| p-value  between the 2 sets of primers | | 0.0037 | 0.156 | 0.0027 | 0.2237 | 0.0264 | 0.0853 | 0.3469 | 0.1405 | 0.0436 | 0.7064 | 0.8508 | 0.3259 |
| Significance (<0.05) | | ** | ns | ** | ns | * | ns | ns | ns | * | ns | ns | ns |
| **Template** | | **Tel-36B4-ds** | | | | | | | | | | | |
| **Primer**  **pair** | tel1/tel2 | 1.76±0.05 | 1.82±0.02 | 1.83±0.02 | 1.77±0.03 | 1.82±0.02 | 1.84±0.02 | 1.77±0.03 | 1.82±0.02 | 1.83±0.01 | 1.78±0.02 | 1.82±0.02 | 1.83±0.03 |
|  | tel1b/tel2b | 1.78±0.02 | 1.82±0.01 | 1.85±0.02 | 1.78±0.02 | 1.83±0.01 | 1.84±0.03 | 1.79±0.03 | 1.83±0.01 | 1.84±0.01 | 1.78±0.01 | 1.82±0.01 | 1.83±0.01 |
|  | telg/telc | 1.77±0.02 | 1.81±0.02 | 1.82±0.02 | 1.79±0.03 | 1.81±0.01 | 1.83±0.04 | 1.79±0.02 | 1.82±0.02 | 1.84±0.02 | 1.79±0.03 | 1.81±0.02 | 1.82±0.02 |
|  | 36B4 | 1.78±0.04 | 1.82±0.02 | 1.82±0.02 | 1.79±0.06 | 1.81±0.02 | 1.83±0.02 | 1.80±0.03 | 1.81±0.02 | 1.82±0.02 | 1.81±0.05 | 1.82±0.02 | 1.84±0.03 |
| p-value  among the 4 sets of primers | | 0.5922 | 0.3451 | 0.0246 | 0.34249 | 0.0331 | 0.9823 | 0.0888 | 0.1359 | 0.0008 | 0.2059 | 0.2746 | 0.3936 |
| Significance (<0.05) | | ns | ns | * | ns | * | ns | ns | ns | *** | ns | ns | ns |
| **Template** | | **Tel-IFNB1-ds** | | | | | | | | | | | |
| **Primer**  **pair** | tel1/tel2 | 1.78±0.05 | 1.81±0.03 | 1.84±0.02 | 1.78±0.03 | 1.81±0.02 | 1.83±0.03 | 1.78±0.05 | 1.82±0.03 | 1.85±0.03 | 1.77±0.05 | 1.80±0.03 | 1.82±0.03 |
|  | tel1b/tel2b | 1.81±0.03 | 1.83±0.02 | 1.85±0.03 | 1.78±0.01 | 1.84±0.02 | 1.85±0.02 | 1.78±0.03 | 1.84±0.02 | 1.87±0.03 | 1.78±0.03 | 1.83±0.02 | 1.84±0.03 |
|  | telg/telc | 1.79±0.03 | 1.81±0.04 | 1.83±0.02 | 1.79±0.03 | 1.81±0.02 | 1.85±0.04 | 1.76±0.03 | 1.80±0.02 | 1.83±0.02 | 1.78±0.02 | 1.81±0.02 | 1.82±0.02 |
|  | IFNB1 | 1.78±0.02 | 1.81±0.02 | 1.82±0.03 | 1.79±0.04 | 1.80±0.02 | 1.83±0.02 | 1.80±0.03 | 1.80±0.02 | 1.82±0.03 | 1.80±0.04 | 1.82±0.03 | 1.81±0.02 |
| p-value  among the 4 sets of primers | | 0.3121 | 0.4591 | 0.1562 | 0.8589 | 0.0168 | 0.167 | 0.2718 | 0.0048 | 0.0051 | 0.4106 | 0.275 | 0.0435 |
| Significance (<0.05) | | ns | ns | * | ns | * | ns | ns | ** | ** | ns | ns | * |
| **Template** | | **Human cell line C1 gDNA** | | | | | | | | | | | |
| **Primer**  **pair** | tel1/tel2 | 1.82±0.06 | 1.85±0.04 | 1.86±0.04 | 1.86±0.06 | 1.85±0.04 | 1.85±0.03 | 1.83±0.05 | 1.83±0.05 | 1.86±0.03 | 1.82±0.04 | 1.86±0.05 | 1.86±0.03 |
|  | tel1b/tel2b | 1.83±0.06 | 1.90±0.07 | 1.89±0.05 | 1.84±0.05 | 1.87±0.03 | 1.89±0.04 | 1.82±0.05 | 1.86±0.03 | 1.93±0.04 | 1.80±0.04 | 1.87±0.01 | 1.87±0.03 |
|  | telg/telc | 1.87±0.04 | 1.85±0.03 | 1.88±0.03 | 1.85±0.05 | 1.86±0.04 | 1.89±0.03 | 1.85±0.07 | 1.90±0.04 | 1.89±0.04 | 1.86±0.03 | 1.87±0.03 | 1.87±0.03 |
|  | 36B4 | 1.91±0.07 | 1.90±0.04 | 1.90±0.04 | 1.90±0.09 | 1.91±0.06 | 1.88±0.02 | 1.91±0.03 | 1.88±0.06 | 1.91±0.03 | 1.88±0.05 | 1.91±0.04 | 1.91±0.05 |
|  | IFNB1 | 1.90±0.04 | 1.87±0.03 | 1.88±0.03 | 1.87±0.06 | 1.87±0.05 | 1.87±0.03 | 1.91±0.10 | 1.87±0.03 | 1.91±0.06 | 1.91±0.06 | 1.85±0.05 | 1.87±0.03 |
| p-value  among the 5 sets of primers | | 0.0411 | 0.1731 | 0.4067 | 0.4781 | 0.1507 | 0.0841 | 0.051 | 0.0675 | 0.0606 | 0.0042 | 0.101 | 0.2047 |
| Significance (<0.05) | | * | ns | ns | ns | ns | ns | ns | ns | ns | ** | ns | ns |
| **Template** | | **Human cell line C3 gDNA** | | | | | | | | | | | |
| **Primer**  **pair** | tel1/tel2 | 1.83±0.07 | 1.89±0.04 | 1.86±0.06 | 1.88±0.06 | 1.82±0.03 | 1.88±0.05 | 1.82±0.05 | 1.88±0.04 | 1.89±0.06 | 1.79±0.06 | 1.90±0.05 | 1.89±0.06 |
|  | tel1b/tel2b | 1.80±0.04 | 1.84±0.04 | 1.86±0.02 | 1.85±0.06 | 1.80±0.01 | 1.89±0.06 | 1.80±0.06 | 1.88±0.06 | 1.88±0.04 | 1.77±0.06 | 1.83±0.04 | 1.86±0.05 |
|  | telg/telc | 1.88±0.04 | 1.88±0.05 | 1.89±0.06 | 1.87±0.07 | 1.86±0.01 | 1.89±0.07 | 1.90±0.04 | 1.88±0.06 | 1.90±0.06 | 1.87±0.05 | 1.88±0.05 | 1.90±0.05 |
|  | 36B4 | 1.94±0.04 | 1.90±0.06 | 1.92±0.05 | 1.93±0.09 | 1.86±0.03 | 1.89±0.03 | 1.98±0.09 | 1.91±0.05 | 1.94±0.07 | 1.87±0.12 | 1.90±0.04 | 1.89±0.04 |
|  | IFNB1 | 1.92±0.08 | 1.88±0.06 | 1.91±0.05 | 1.87±0.08 | 1.85±0.02 | 1.87±0.06 | 1.89±0.07 | 1.89±0.03 | 1.90±0.07 | 1.92±0.07 | 1.89±0.04 | 1.90±0.03 |
| p-value  among the 5 sets of primers | | 0.0033 | 0.2353 | 0.148 | 0.5356 | 0.0003 | 0.976 | 0.0008 | 0.7992 | 0.5143 | 0.0305 | 0.0512 | 0.74 |
| Significance (<0.05) | | ** | ns | ns | ns | ** | ns | *** | ns | ns | * | ns | ns |
| **Primer concentration (nM)** | | **100** | **500** | **900** | **100** | **500** | **900** | **100** | **500** | **900** | **100** | **500** | **900** |
| **Cycling program** | | **#1 (60^o^C only)** | | | **#2 (49^o^C -60^o^C)** | | | **#3 (56^o^C -60^o^C)** | | | **#4 (60^o^C -60^o^C)** | | |

ns: not significant; *: p-value <0.05; **: p-value <0.01; ***: p-value <0.001. “2 sets of primers”: 36B4 and IFNB1. “3 sets of primers”: tel1/tel2, tel1b/tel2b and telg/telc. “4 sets of primers”: tel1/tel2, tel1b/tel2b, telg/telc and 36B4 or tel1/tel2, tel1b/tel2b, telg/telc and IFNB1. “5 sets of primers”: tel1/tel2, tel1b/tel2b, telg/telc, 36B4 and IFNB1.
