# Supplementary material for: A novel metric to improve mismatched primer selection and quantification accuracy in amplifying DNA repeats for quantitative polymerase chain reactions
Source: PLoS One. 2023 Oct 9;18(10):e0292559. doi: 10.1371/journal.pone.0292559 (PMC10561853; doi:10.1371/journal.pone.0292559)
Supplement: S3 Table — E values are calculated from the corresponding standard curves in S1 Fig and N0 values are calculated from basic PCR kinetic formula. Cq values are median from triplicate wells corresponding to the qPCR data in S1 Fig. (DOCX) [file pone.0292559.s004.docx]

**Supplemental Table 3. An example of N_0_ and *E* values calculated with the traditional method for reactions containing 6 pg input template using Cycling Program #1 (60^o^C only).** *E* values were calculated from the corresponding standard curves in Supplemental Fig 1 and N_0_ values were calculated from basic PCR kinetic formula. C_q_ values were median from triplicate wells corresponding to the qPCR data in Supplemental Fig 1.

1. **C_q_ threshold set at 0.2**

| **Primer concentration (nM)** | | **100** | | | **500** | | | **900** | | |
| --- | --- | --- | --- | --- | --- | --- | --- | --- | --- | --- |
| **Template** | **Primer pair** | **N_0_** | **C_q_** | ***E*** | **N_0_** | **C_q_** | ***E*** | **N_0_** | **C_q_** | ***E*** |
| Tel-ds | tel1/tel2 | 4.00 x 10^-8^ | 24.23 | 1.89 | 3.29 x 10^-7^ | 20.58 | 1.91 | 2.43 x 10^-7^ | 20.24 | 1.96 |
|  | tel1b/tel2b | 2.94 x 10^-6^ | 17.48 | 1.89 | 4.72 x 10^-5^ | 13.23 | 1.88 | 3.70 x 10^-5^ | 12.97 | 1.94 |
|  | telg/telc | 5.52 x 10^-9^ | 25.67 | 1.97 | 2.09 x 10^-7^ | 20.78 | 1.94 | 1.27 x 10^-7^ | 20.44 | 2.01 |
| 36B4-ds | 36B4 | 3.71 x 10^-4^ | 9.14 | 1.99 | 9.51 x 10^-4^ | 8.20 | 1.92 | 3.54 x 10^-4^ | 8.95 | 2.03 |
| IFNB1-ds | IFNB1 | 3.87 x 10^-4^ | 9.08 | 1.99 | 6.26 x 10^-4^ | 8.27 | 2.01 | 5.26 x 10^-4^ | 8.51 | 2.01 |

1. **Automatic C_q_ threshold set by the software**

| **Primer concentration (nM)** | | **100** | | | **500** | | | **900** | | |
| --- | --- | --- | --- | --- | --- | --- | --- | --- | --- | --- |
| **Template** | **Primer pair** | **N_0_** | **C_q_** | ***E*** | **N_0_** | **C_q_** | ***E*** | **N_0_** | **C_q_** | ***E*** |
| Tel-ds | tel1/tel2 | 3.84 x 10^-8^ | 23.34 | 1.89 | 3.70 x 10^-7^ | 20.94 | 1.90 | 2.14 x 10^-7^ | 21.40 | 1.97 |
|  | tel1b/tel2b | 2.94 x 10^-6^ | 17.74 | 1.89 | 5.44 x 10^-5^ | 14.64 | 1.87 | 3.34 x 10^-5^ | 14.61 | 1.95 |
|  | telg/telc | 5.52 x 10^-9^ | 25.54 | 1.97 | 2.32 x 10^-7^ | 21.70 | 1.93 | 1.40 x 10^-7^ | 21.25 | 2.00 |
| 36B4-ds | 36B4 | 3.74 x 10^-4^ | 9.03 | 1.99 | 9.61 x 10^-4^ | 9.41 | 1.92 | 3.33x 10^-4^ | 10.00 | 2.03 |
| IFNB1-ds | IFNB1 | 3.87 x 10^-4^ | 9.07 | 1.99 | 6.23 x 10^-4^ | 9.43 | 2.00 | 5.38 x 10^-4^ | 9.43 | 2.00 |
