# Supplementary material for: A novel metric to improve mismatched primer selection and quantification accuracy in amplifying DNA repeats for quantitative polymerase chain reactions
Source: PLoS One. 2023 Oct 9;18(10):e0292559. doi: 10.1371/journal.pone.0292559 (PMC10561853; doi:10.1371/journal.pone.0292559)
Supplement: S5 Table — (DOCX) [file pone.0292559.s006.docx]

**Supplemental Table 5. Statistical tests of ratio of amplification efficacy (mean±SD) of (*f*_(tel)_/*f*_(36B4)_ or *f*_(tel)_/*f*_(IFNB1)_) in Table 6 between reactions using Cycling Program #2 or #3 in compared to #4.**

| **Primer**  **concentration**  **(nM)** | **Template** | **Tel-36B4-ds** | | | **Tel-IFNB1-ds** | | |
| --- | --- | --- | --- | --- | --- | --- | --- |
|  | **Cycling program** | $\frac{\boldsymbol{f}_{\left( \boldsymbol{tel}\boldsymbol{1} \right)}}{\boldsymbol{f}_{\left( \boldsymbol{36}\boldsymbol{B}\boldsymbol{4} \right)}}$ | $\frac{\boldsymbol{f}_{\left( \boldsymbol{tel}\boldsymbol{1}\boldsymbol{b} \right)}}{\boldsymbol{f}_{\left( \boldsymbol{36}\boldsymbol{B}\boldsymbol{4} \right)}}$ | $\frac{\boldsymbol{f}_{\left( \boldsymbol{telg} \right)}}{\boldsymbol{f}_{\left( \boldsymbol{36}\boldsymbol{B}\boldsymbol{4} \right)}}$ | $\frac{\boldsymbol{f}_{\left( \boldsymbol{tel}\boldsymbol{1} \right)}}{\boldsymbol{f}_{\left( \boldsymbol{IFNB}\boldsymbol{1} \right)}}$ | $\frac{\boldsymbol{f}_{\left( \boldsymbol{tel}\boldsymbol{1}\boldsymbol{b} \right)}}{\boldsymbol{f}_{\left( \boldsymbol{IFNB}\boldsymbol{1} \right)}}$ | $\frac{\boldsymbol{f}_{\left( \boldsymbol{telg} \right)}}{\boldsymbol{f}_{\left( \boldsymbol{IFNB}\boldsymbol{1} \right)}}$ |
| **100 nM** | #2 (49^o^C-60^o^C) | (1.85±0.15) x 10^-3^ | (4.26±1.50) x 10^-2^ | (2.35±0.35) x 10^-3^ | (3.30±0.48) x 10^-3^ | (6.04±0.38) x 10^-2^ | (2.36±0.24) x 10^-3^ |
|  | #3 (56^o^C-60^o^C) | (1.50±0.20) x 10^-3^ | (1.50±0.22) x 10^-1^ | (4.41±0.81) x 10^-3^ | (2.00±0.29) x 10^-3^ | (1.36±0.20) x 10^-1^ | (3.16±0.65) x 10^-3^ |
|  | #4 (60^o^C-60^o^C) | (2.33±1.52) x 10^-4^ | (9.70±1.08) x 10^-3^ | (8.29 ±7.74) x 10^-5^ | (1.25±0.16) x 10^-4^ | (7.57±0.50) x 10^-3^ | (3.06±1.07) x 10^-5^ |
|  | p-value between #2 and #4 | <0.0001 | 0.0002 | <0.0001 | <0.0001 | <0.0001 | <0.0001 |
|  | Significance (<0.05) | **** | *** | **** | **** | **** | **** |
|  | p-value between #3 and #4 | <0.0001 | <0.0001 | <0.0001 | <0.0001 | <0.0001 | <0.0001 |
|  | Significance (<0.05) | **** | **** | **** | **** | **** | **** |
| **500 nM** | #2 (49^o^C-60^o^C) | (3.63±0.85) x 10^-3^ | (9.08±1.35) x 10^-2^ | (6.52±2.07) x 10^-3^ | (5.41±0.33) x 10^-3^ | (1.09±0.06) x 10^-1^ | (4.65±0.45) x 10^-3^ |
|  | #3 (56^o^C-60^o^C) | (3.85±1.90) x 10^-3^ | (4.07±0.80) x 10^-1^ | (2.91±0.73) x 10^-2^ | (5.14±0.28) x 10^-3^ | (3.60±0.24) x 10^-1^ | (1.53±0.08) x 10^-2^ |
|  | #4 (60^o^C-60^o^C) | (1.54±2.24) x 10^-3^ | (6.23±1.15) x 10^-2^ | (1.43±1.88) x 10^-3^ | (7.12±0.72) x 10^-4^ | (5.30±0.79) x 10^-2^ | (5.04±1.42) x 10^-4^ |
|  | p-value between #2 and #4 | 0.025 | 0.0002 | <0.0001 | <0.0001 | <0.0001 | <0.0001 |
|  | Significance (<0.05) | * | *** | **** | **** | **** | **** |
|  | p-value between #3 and #4 | 0.0317 | <0.0001 | <0.0001 | <0.0001 | <0.0001 | <0.0001 |
|  | Significance (<0.05) | * | **** | **** | **** | **** | **** |
| **900 nM** | #2 (49^o^C-60^o^C) | (6.34±0.60) x 10^-3^ | (1.34±0.45) x 10^-1^ | (1.03±0.21) x 10^-2^ | (9.44±0.58) x 10^-3^ | (1.54±0.22) x 10^-1^ | (6.53±0.38) x 10^-3^ |
|  | #3 (56^o^C-60^o^C) | (6.34±1.71) x 10^-3^ | (4.06±0.81) x 10^-1^ | (4.45±1.03) x 10^-2^ | (9.20±1.10) x 10^-3^ | (4.29±0.50) x 10^-1^ | (2.20±0.21) x 10^-2^ |
|  | #4 (60^o^C-60^o^C) | (1.13±0.12) x 10^-3^ | (1.10±0.20) x 10^-1^ | (1.15±0.24) x 10^-3^ | (9.30±1.65) x 10^-4^ | (8.19±1.19) x 10^-2^ | (7.97±3.43) x 10^-4^ |
|  | p-value between #2 and #4 | <0.0001 | 0.1659 | <0.0001 | <0.0001 | <0.0001 | <0.0001 |
|  | Significance (<0.05) | **** | ns | **** | **** | **** | **** |
|  | p-value between #3 and #4 | <0.0001 | <0.0001 | <0.0001 | <0.0001 | <0.0001 | <0.0001 |
|  | Significance (<0.05) | **** | **** | **** | **** | **** | **** |

ns: not significant; *: p-value <0.05; **: p-value <0.01; ***: p-value <0.001, ****: p-value <0.0001.
